# Kinase activities in pancreatic ductal adenocarcinoma with prognostic and therapeutic avenues

**DOI:** 10.1002/1878-0261.13625

**Published:** 2024-04-22

**Authors:** Andrea Vallés‐Martí, Richard R. de Goeij‐de Haas, Alex A. Henneman, Sander R. Piersma, Thang V. Pham, Jaco C. Knol, Joanne Verheij, Frederike Dijk, Hans Halfwerk, Elisa Giovannetti, Connie R. Jiménez, Maarten F. Bijlsma

**Affiliations:** ^1^ Department of Medical Oncology, Amsterdam University Medical Center VU University Amsterdam The Netherlands; ^2^ OncoProteomics Laboratory Cancer Center Amsterdam The Netherlands; ^3^ Cancer Biology Cancer Center Amsterdam The Netherlands; ^4^ Pharmacology Laboratory Cancer Center Amsterdam The Netherlands; ^5^ Department of Pathology Amsterdam University Medical Center The Netherlands; ^6^ Cancer Pharmacology Lab, AIRC Start‐Up Unit Fondazione Pisana per la Scienza San Giuliano Terme Italy; ^7^ Laboratory for Experimental Oncology and Radiobiology, Center for Experimental and Molecular Medicine, Amsterdam University Medical Center University of Amsterdam The Netherlands

**Keywords:** kinase activity, pancreatic ductal adenocarcinoma, personalized medicine, phosphoproteome

## Abstract

Pancreatic ductal adenocarcinoma (PDAC) is a devastating disease with a limited number of known driver mutations but considerable cancer cell heterogeneity. Phosphoproteomics provides a direct read‐out of aberrant signaling and the resultant clinically relevant phenotype. Mass spectrometry (MS)‐based proteomics and phosphoproteomics were applied to 42 PDAC tumors. Data encompassed over 19 936 phosphoserine or phosphothreonine (pS/T; in 5412 phosphoproteins) and 1208 phosphotyrosine (pY; in 501 phosphoproteins) sites and a total of 3756 proteins. Proteome data identified three distinct subtypes with tumor intrinsic and stromal features. Subsequently, three phospho‐subtypes were apparent: two tumor intrinsic (Phos1/2) and one stromal (Phos3), resembling known PDAC molecular subtypes. Kinase activity was analyzed by the Integrative iNferred Kinase Activity (INKA) scoring. Phospho‐subtypes displayed differential phosphorylation signals and kinase activity, such as FGR and GSK3 activation in Phos1, SRC kinase family and EPHA2 in Phos2, and EGFR, INSR, MET, ABL1, HIPK1, JAK, and PRKCD in Phos3. Kinase activity analysis of an external PDAC cohort supported our findings and underscored the importance of PI3K/AKT and ERK pathways, among others. Interestingly, unfavorable patient prognosis correlated with higher RTK, PAK2, STK10, and CDK7 activity and high proliferation, whereas long survival was associated with MYLK and PTK6 activity, which was previously unknown. Subtype‐associated activity profiles can guide therapeutic combination approaches in tumor and stroma‐enriched tissues, and emphasize the critical role of parallel signaling pathways. In addition, kinase activity profiling identifies potential disease markers with prognostic significance.

## Graphical abstract

Despite the treatment advancements in pancreatic ductal adenocarcinoma, 5‐year survival rates are still below 10%. Analyzing the phosphoproteome of 42 tumors, three subtypes with intrinsic and stromal features were revealed, each displaying kinase activity patterns with potential treatment options. Kinase activities associated with specific mutations, and correlated with clinical prognosis. This study provides insights for tailored and combination strategies.

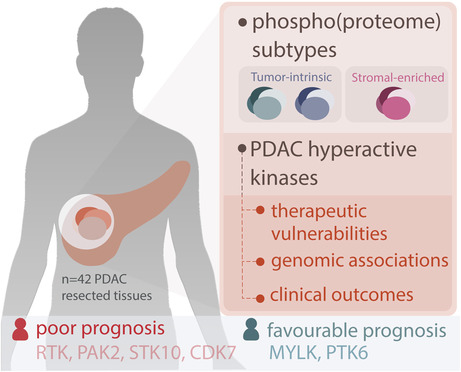

**Abbreviations**
CAF: cancer‐associated fibroblast
EMT: epithelial‐to‐mesenchymal transition
GSEA: gene set enrichment analysis
IMAC: immobilized metal affinity chromatography
INKA: integrative iNferred kinase activity
LC–MS/MS: liquid chromatography‐mass spectrometry
LFQ: label free quantitation
OCT: optimal cutting temperature
OS: overall survival
PDAC: pancreatic ductal adenocarcinoma
pST: phosphoserine/phosphothreonine
PTM‐SEA: post‐translational modification signature enrichment analysis
pY IP: phosphotyrosine immunoprecipitation
pY: phosphotyrosine
WT: wildtype

## 1 Introduction

Due to a late diagnosis, disease heterogeneity, and a lack of effective treatments, pancreatic cancer 5‐year survival rates are still low, typically below 10% [1]. Also, its climbing incidence has led to the prediction that by 2030 pancreatic cancer will be a top‐3 ranking cause of cancer death together with lung and liver cancers [2]. Survival of patients with surgically resectable tumors has improved over the last decade. However, pancreatic ductal adenocarcinoma (PDAC) tumors still remain difficult to treat, particularly in the locally advanced and metastatic setting [3, 4].

Transcriptomics has revealed relevant PDAC molecular subtypes [5], but has yet to predict effective treatments. The complementary information provided by the combination of genomic and proteomic analyses, the latest integrating genetic and epigenetic changes, is believed to have a major role in drug target discovery [6]. However, genetic profiling has revealed a very limited set of actionable mutations in PDAC, including the relative efficacy of oxaliplatin against germline‐mutated *BRCA1/2* tumors. Recent studies have also tried to establish a correlation between genetic aberrations and clinical survival, with limited success [7, 8, 9]. This is partially due to the substantial genetic variation between PDAC tumors at other omics levels [10] and cell populations [11], as well as the low tumor cellularity and the complex relationship with the tumor microenvironment [12].

Pancreatic ductal adenocarcinoma exhibits a high prevalence of activated *KRAS*, a key player in signal transduction that was considered undruggable until recently [13]. Despite the development of KRAS inhibitors, resistance may remain a challenge [14]. This makes phosphoproteomics a useful approach to measure the associated hyperactive pathways that could help to unravel effective drug combination strategies. Several multi‐omic studies have unraveled drug vulnerabilities by exploring proteomics and phosphoproteomics in patients with colorectal [15], lung [16], esophageal [17], gastric [18], breast [19], and leukemic [20, 21] tumors, and also very recently in PDAC [22, 23, 24, 25]. A MS‐based approach allows to identify and quantify phosphorylation sites in proteins that are involved in oncogenic cellular processes. By quantifying the relative abundance of these proteins and the kinase–substrate relationships, associated biology and drug targets can be revealed. Here, we charted the phosphoproteome of 42 PDAC tumors to reveal signaling pathways that may be involved in PDAC development and progression, as well as to validate kinase targets recently identified in preclinical studies [26, 27] that could be exploited for therapeutic intervention.

## 2 Materials and methods

### 2.1 Clinical samples

Tumor patient samples from pancreaticoduodenectomies, which were part of a previously collected PDAC cohort for RNA‐sequencing [28], were evaluated for phosphoproteome profiling. Tumor tissue specimens, snap‐frozen in liquid nitrogen and stored at −80 °C, were retrospectively collected from the tissue archive of the Department of Pathology (approved by the Committee Review Biobanks, CTB.2015‐081), with the understanding and written consent of each subject, for the purpose of phosphoproteomic research. Retrospective collection was conducted at the Academic Medical Center location of Amsterdam UMC in accordance with ethical guidelines of Amsterdam UMC Research Code, based on the “Code of conduct Health Research” (Dutch Committee on Regulation of Health Research) between September 2019 and August 2020. The study methodologies were in accordance with the guidelines established by the Declaration of Helsinki. Clinicopathological data were obtained through the department of Pathology (Table S1).

### 2.2 Protein isolation from clinical samples

Feasibility of performing a phosphoproteome profiling of the remaining tissue was evaluated by assessing tumor volume and estimating protein input to achieve at least 2 mg of protein. This was equivalent to 20 slices of 10 μm thickness from a 1‐cm^2^ area tissue block, which will give a tissue wet weight of 20 mg. Based on this criterion, a total of 42 tissues were selected. Tumor tissues were optimal cutting temperature (OCT)‐embedded, which interferes with the mass spectrometer [29]. Therefore, an OCT removal prior to MS‐based proteomics was performed as previously described [29, 30]. First, big OCT pieces were carefully removed with a razorblade inside the cryotome at −20 °C. Per tumor sample, fifty to sixty 20 μm cryo‐slices were collected in a previously weighted 2‐mL vial and tissue wet weight was estimated. Samples were washed by vortexing and centrifuging through a series of 75% ethanol, H_2_O and 100% ethanol. Then, tumor slices were lysed for phospho‐protein extraction by adding 2 mL urea lysis buffer followed by three cycles of sonication (20 s on, 20 s off at maximum amplitude). Samples were then centrifuged (16 000 ***g***, 10 min, 10 °C) and supernatant was collected. Protein concentration was determined with a Pierce BCA Protein Assay Kit (Thermo Scientific, Breda, The Netherlands), and all samples were stored at −80 °C until further use.

### 2.3 Western blotting

Sample processing and evaluation of phosphotyrosine was performed as previously described [26]. Phosphotyrosine levels were evaluated prior to sample processing by western blotting. Protein samples were denatured in sample running buffer (65% MiliQ, 25% NuPAGE™ LDS Sample Buffer (4×), 10% dithiothreitol (DTT) 1 m) and heated 3 min at 95 °C and immediately loaded in 4–12% polyacrylamide gels (Invitrogen™ NuPAGE™, Thermo Scientific) which were run at 150 V for 90 min. Proteins were then transferred to a nitrocellulose membrane using 100 V for 2 h. To ascertain equal protein loading, Ponceau staining was used at this step. Membranes were then incubated in blocking solution (5% bovine serum albumin in PBS‐0.1% Tween20 (cat #003005; Thermo Fisher, Bremen, Germany) (PBS‐T)) for 1 h. After blocking, nitrocellulose membranes were incubated with the primary antibody anti‐P‐Tyr‐1000 (cat #8954; Cell Signaling Technology, Leiden, The Netherlands) overnight at 4 °C. Then, membranes were washed five times in PBS‐T for 5 min and incubated for 1 h at room temperature with secondary antibody anti‐rabbit IgG, HRP‐linked Antibody (cat #7074; Cell Signaling Technology). Membranes were again washed five times in PBS‐T for 5 min. Blots were then incubated for 5 min in chemiluminescence substrate (SuperSignal™ West Pico PLUS cat #34580, Thermo Scientific) and finally developed using the UVITEC imaging system (Cambridge, UK).

### 2.4 Sample preparation for phosphotyrosine enrichment

Sample processing and evaluation of phosphotyrosine was performed as previously described [26]. For PDAC analyses, a total of 42 PDAC tissues were analyzed. To boost identifications, we included two replicates of equiproportional pooled lysates of five PDAC cell lines (PANC‐1, Suit‐2, CFPAC‐1, HPAC, Mia PaCa‐2). A control lysate of HCT116 (colon carcinoma cell line) was used as workflow control. We also included two PDAC tissues in duplicate (samples #16, #53 collected in 2 vials cryocutting). To assess tumor heterogeneity, we included two different tumor pieces from the same patient (Samples #1 and #9).

For phosphotyrosine immunoprecipitation (pY‐IP) 2.2–5 mg protein was used. Cryostat sections of 20 μm were lysed in 9 m ureabuffer (9 m urea/20 mm HEPES (pH 8) lysis buffer containing 1 mm sodium orthovanadate, 2.5 mm sodium pyrophosphate, and 1 mm β‐glycerophosphate). Upon lysis, proteins were reduced with DTT (dithiothreitol, 5 mm) and alkylated with IAA (iodoacetamide, 10 mm) followed, by dilution to 2 m urea. Samples were digested with trypsin (Promega, Leiden, The Netherlands) overnight (1 : 100 ratio) and thereafter acidified by addition of TFA (1% end concentration). The digests were desalted using Oasis HLB columns (500 mg capacity; Waters, Milford, MA, USA), eluted in 60% ACN/0.1% TFA and lyophilized for 48 h in a freeze dryer (Christ alfa 2–4 LSC).

Lyophilized phosphopeptides were dissolved in IAP buffer (50 mm MOPS/NaOH pH 7.2, 10 mm Na_2_HPO_4_, 50 mm NaCl) and incubated with PTMScan pY antibody‐conjugated beads (p‐Tyr‐1000) (Cell Signaling Technology) at a ratio of 4 μL slurry per mg protein at 4 °C for 2 h, as previously described [31]. Then, after washing with cold IAP buffer and Milli‐Q water, peptides were eluted from the beads in two steps with 0.15% TFA. Phosphopeptides were desalted using a 200‐μL STAGE tip fitted with a 1 mm‐needle punch of SDB‐XC SPE material at the narrow end. The SDB‐XC bed was activated with 20 μL of 80% ACN/0.1% TFA and equilibrated with 20 μL of 0.1% TFA. Phosphopeptides were loaded and centrifuged for 3 min at 1000 ***g***. SDB‐XC beds were then washed with 20 μL of 0.1% TFA, and desalted phosphopeptides were eluted with 20 μL of 80% ACN/0.1% TFA. Phosphopeptides were dried in a vacuum centrifuge and dissolved in 20 μL of 4% ACN/0.5% TFA prior to injection and stored at 4 °C until LC–MS/MS measurement on the same day.

### 2.5 Sample preparation for global phosphoproteomics (pS/pT) enrichment

Phosphoproteomic enrichment was performed as previously described [26]. In brief, non‐bound fractions of the pY IP‐samples were used for global phosphoproteomics using (Fe(III)‐NTA cartridges) on the BRAVO platform (Agilent, Amstelveen, The Netherlands) using Phospho Enrichment v2.0 protocol as previously described [32]. A total of 200 μg peptides of the non‐bound fraction for each sample were desalted using a 10 mg OASIS HLB cartridge and eluted in 80% ACN/0.1% TFA solution. The cartridges were primed with 100% ACN/0.1% TFA, equilibrated with 80% ACN/0.1% TFA, whereafter the samples were loaded and washed with 80% ACN/0.1% TFA. The phosphopeptides were eluted in 25 μL of 5% NH_4_OH/30% ACN and then transferred to glass‐lined autosampler vials and dried in a vacuum centrifuge at 45 °C. Finally, phosphopeptides were dissolved in 20 μL of 4% ACN/0.5% TFA prior to injection in the LC–MS.

### 2.6 Data acquisition by LC–MS/MS

Data acquisition was performed as previously described [26]. Peptides were separated on an Ultimate 3000 nanoLC–MS/MS system (Dionex LC‐Packings, Amsterdam, the Netherlands) equipped with a 50‐cm 75 μm ID C18 Acclaim pepmap column (Thermo Scientific). After injection, peptides were trapped at 3 μL·min^−1^ on a 10‐mm, 75‐μm ID Acclaim Pepmap trap column (Thermo Scientific) in buffer A (buffer A: 0.1% formic acid, buffer B: 80% ACN/0.1% formic acid), and separated at 300 mL·min^−1^ with a 10–40% buffer B gradient in 90 min (120 min inject‐to‐inject). Eluting peptides were ionized at a potential of +2 kV and introduced into a Q Exactive HF mass spectrometer (Thermo Fisher). Intact masses were measured in the orbitrap with a resolution of 120 000 (at *m*/*z* 200) using an automatic gain control (AGC) target value of 3 × 10^6^ charges. Peptides with the top 15 highest signals (charge states 2+ and higher) were submitted to MS/MS in the higher‐energy collision cell (1.6‐Da isolation width, 25% normalized collision energy). MS/MS spectra were acquired in the Orbitrap with a resolution of 15k (at *m*/*z* 200) using an AGC target value of 1 × 10^6^ charges and an under fill ratio of 0.1%. Dynamic exclusion was applied with a repeat count of 1 and an exclusion time of 30 s.

### 2.7 Phosphopeptide and ‐site identification and quantification by LC–MS/MS

As previously described [26], MS/MS spectra of immobilized metal affinity chromatography (IMAC) and phosphotyrosine immunoprecipitation (pY IP) phosphopeptide enrichment experiments were searched separately against the Swissprot human FASTA file (downloaded January 2021, canonical and isoforms; 42 383 entries) using maxquant 1.6.10.43 [33]. Enzyme specificity was set to trypsin, and up to two missed cleavages were allowed. Cysteine carboxamidomethylation (+57.021464 Da) was treated as fixed modification and serine, threonine, and tyrosine phosphorylation (+79.966330 Da), methionine oxidation (+15.994915 Da), and N‐terminal acetylation (+42.010565 Da) as variable modifications. Peptide precursor ions were searched with a maximum mass deviation of 4.5 p.p.m. and fragment ions with a maximum mass deviation of 20 p.p.m. Peptide and protein identifications were filtered at a false discovery rate of 1% using a decoy database strategy. The minimal peptide length was set at 7 amino acids, the minimum Andromeda score for modified peptides was 40, and the corresponding minimum delta score was 6. Proteins that could not be differentiated based on MS/MS spectra alone were clustered into protein groups (all default maxquant settings). Phosphopeptide identifications were propagated across samples using the “match between runs” option checked.

### 2.8 Label‐free phosphopeptide quantification

Phosphopeptide quantification was performed as previously described [26]. For phosphopeptide data, we used data from the maxquant “modificationSpecificPeptides” table. For phosphosite (psite) data, we used data from the maxquant “Phospho (STY) Sites” table. Phosphopeptides and phosphosites were quantified from the area under the curve of the MS1 signal of each eluting peptide (“Intensity” in maxquant). For calculation of kinase INKA scores, phosphopeptide MS/MS spectral counts were calculated from the maxquant evidence file using R. IMAC normalization was performed to the median site intensity and pY IP normalization was performed to the total lysate spectral count. Missing value imputation was not performed. Contaminants and all‐zero intensity rows were excluded. Then, data were further analyzed by custom scripts using R (version 4.2.1) [34]. Only phosphosites with a localization probability ≥ 0.75 (class 1) were used for further analysis.

### 2.9 Statistical analyses: data filtering and analysis

Filtering of 10% and 100% data presence (DP) was applied to perform sample‐sample and class I phosphosite‐phosphosite correlation analyses, respectively. Sample‐sample and phosphosite‐phosphosite correlation analyses were based on Pearson correlation and plotted with gplots R package [35]. Pearson correlation of the HCT116 samples was performed between phosphosites identified in all replicates. Phosphosite identifications and tumor cellularity were correlated by Spearman correlation. For consensus clustering analysis we used the consensusclusterplus R package [36]. Analysis was performed on the proteome and phosphoproteome datasets after applying a 10% DP filtering and calculating the median absolute deviation (MAD) to idenfity top 10 most variable identifications. Phosphosite and protein resulting data‐sets were plotted using the complexheatmap R package [37]. Differentially expressed proteins and phosphorylated sites between subtypes, overall survival and mutation status, were identified using limma statistics with ion R package (“https://tvpham.github.io/ion”). Datasets were first processed with a 10% DP filtering. Benjamini–Hochberg adjusted *P* value threshold was set to 0.05 and fold change ≥ 2 or ≤ −2. Single sample gene set enrichment analysis (GSEA) [38] was performed with a pre‐ranked list after multiplying the negative log_10_ *P*‐values derived from limma with the sign of the fold change. We used the gsva R package [39] with a minimum size of 5 genes and a maximum of 1000. Gene signatures were retrieved from Molecular Signatures Database (Human MSigDB v2022.1.Hs) [40]. Enrichment scores were then averaged per protein cluster. Post‐translational modification signature enrichment analysis (PTM‐SEA) [41] was performed using the normalized site intensities. In case of duplicated phosphosite amino acid windows, the most abundant (row sum intensities) entry was used. PTM‐SEA was performed using the GenePattern platform [42]. Kinase activity was inferred from phosphoproteomic data using Integrative iNferred Kinase Activity (INKA) analysis as previously described [31]. INKA pipeline is available at https://inkascore.org/.

## 3 Results

### 3.1 Protein and phosphoprotein landscapes of PDAC tumor tissue

A comprehensive phosphoproteomic analysis was conducted on a set of 42 resected PDAC samples. These samples were derived from a larger study in which transcriptomic data were obtained (Fig. S1a) [28]. Tumor samples were selected based on tissue quantity to achieve at least 2 mg of protein required for pY phosphopeptide enrichment in addition to global phosphopeptide enrichment. The clinical and pathological features of these samples are summarized in Table S1. By using a two‐step phosphopeptide enrichment with phosphotyrosine immunoprecipitation (pY IP) and immobilized metal affinity chromatography (IMAC, global), followed by label‐free MS analysis, we generated a comprehensive dataset of 19 936 pS/T sites, 1208 pY sites and a total of 3756 proteins after data pre‐processing (see Section 2; Figs S1–S4). Underscoring the integrity of our dataset, the five to six workflow replicates of HCT116 cell line external reference samples clustered together in an unsupervised analysis (Fig. S1b–d). Moreover, these reference samples showed robust and consistent class I phosphosite identifications in global and pY (Spearman's correlation coefficient > 0.938 and 0.864, respectively). To ensure pY quality prior to phosphopeptide capture, pY levels were evaluated by western blotting, which showed variance among PDAC samples (Fig. S3h) indicating differences in signaling state. PDAC pS/T site identifications positively correlated with tumor cellularity (10–70%, median 40%), assessed by a pathologist (Fig. S2f), although no significant correlation was found for pY sites and proteins (Figs S3f and S4f, respectively). This suggests that the abundance of stroma may reduce the number and/or type of pS/T sites, in accordance with the elevated and aberrant signaling typically associated with the proliferative tumor cell population.

### 3.2 Proteomic characterization of PDAC tumors unravels distinct tumor biology

**Fig. 1:**
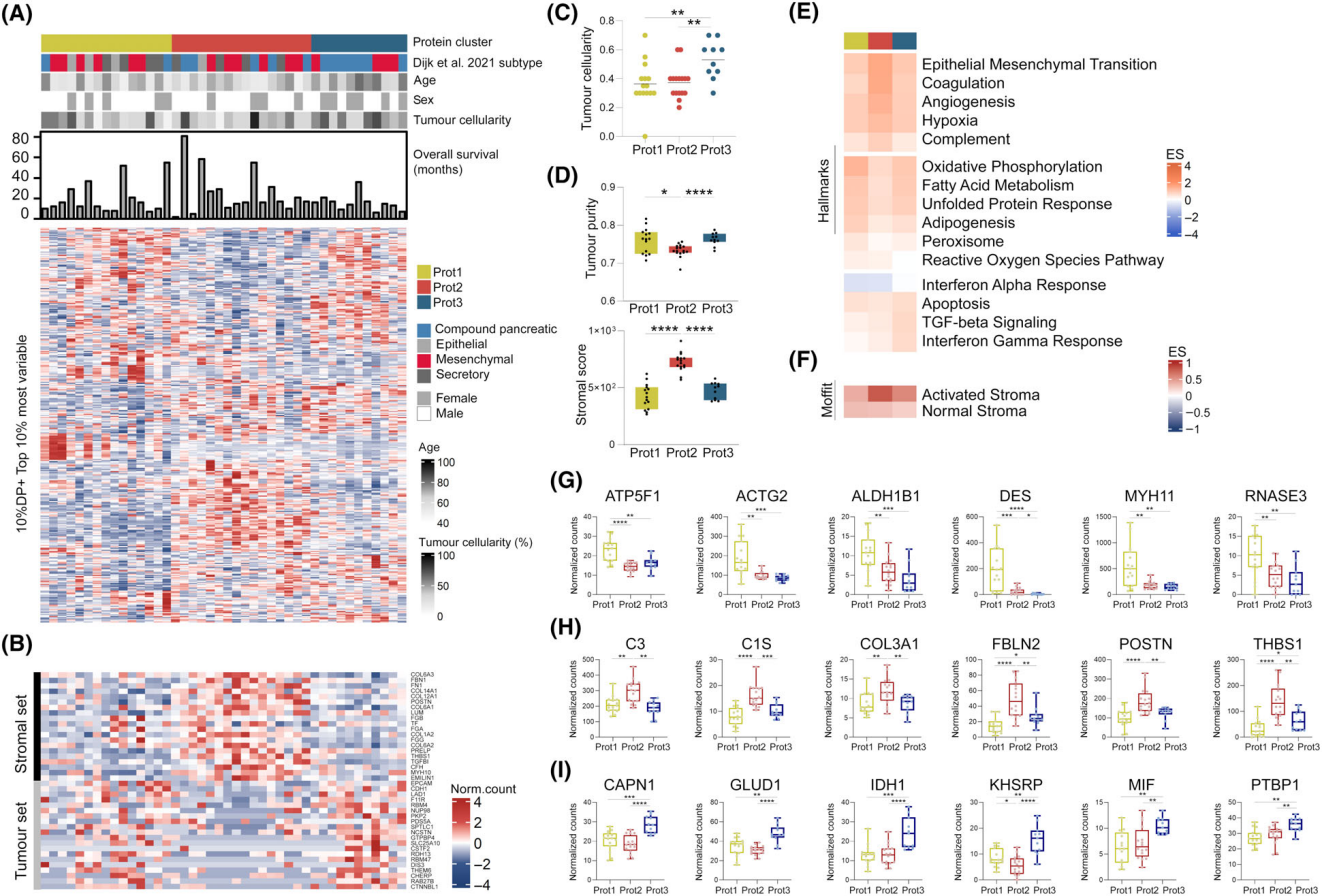
Proteome subtyping of PDAC tumor tissues. (A) Proteome subtypes generated by consensus clustering (see Fig. S5). Hierarchical clustering was built with the normalized protein counts after a 10% DP and top 10% most variable by median absolute deviation (MAD) filtering. Corresponding age, sex, tumor cellularity, overall survival in months, mRNA [28] and protein subtypes were annotated. (B) Enriched stromal and tumor markers from microdissected pancreatic ductal adenocarcinoma (PDAC) proteome by Le Large et al. [46]. (C) Tumor cellularity assessed by a pathologist per protein cluster, significantly different by Kruskal–Wallis test, *P* = 0.0072. Two group comparisons tested by Mann–Whitney *t*‐test. (D) ESTIMATE‐based tumor purity per protein cluster, significantly different by Kruskal–Wallis test, *P* = 0.0257, and ESTIMATE‐based stromal content per protein cluster, significantly different by Kruskal–Wallis test, *P* < 0.0001. Two group comparisons tested by Mann–Whitney test. (E, F) Single‐sample gene set enrichment analysis (ssGSEA) averaged enrichment score (ES) of signatures Hallmarks and Moffit activated and normal stroma [43]. (G–I) Example of proteins differentially expressed with *P* ≤ 0.05 in Prot1 cluster, Prot2 cluster, and Prot3 cluster. Significance was performed using non‐parametric Kruskal–Wallis test (three group comparison) and Mann–Whitney test (two group comparison). See also Figs S4 and S5, and Table S2. *P < 0.05, **P < 0.01, ***P < 0.001, ****P < 0.0001.

A large number of transcriptomic studies have interrogated PDAC heterogeneity and identified distinct subtypes [5, 28, 43, 44, 45]. To investigate the potential association between RNA and protein‐based subtypes, we studied the proteomic features of a subset (42/90) of the tissue cohort from Dijk et al. [28], in which four transcriptomic subtypes were identified. We then grouped tumors by consensus clustering into three main protein subtypes (Prot1‐3) using the top 10% most variable proteins (Fig. 1, Fig. S5a) [36]. Using the physically separated tumor and stromal proteomics profiles obtained in a recent laser microdissection effort [46] and by ESTIMATE analysis, Prot1 and Prot3 patient clusters were found to be tumor‐cell associated, whereas Prot2 cluster was enriched for stroma (Fig. 1B–D) [47]. This was further supported by the Prot2 enrichment of the activated stroma signature established by Moffit et al. [43] (Fig. 1F).

These protein‐based subtypes were characterized by distinct biology (Fig. 1E). The stroma‐associated Prot2 cluster was significantly enriched for EMT, coagulation, angiogenesis, and hypoxia signatures. In addition, most of the tumors classified as basal‐like on mRNA were grouped in the Prot2 cluster, indicating that tumor cell‐intrinsic mesenchymal signatures also exist in this subgroup [43]. Moreover, Prot2 showed a mixed profile of mesenchymal, compound and secretory tumors using the Dijk et al.'s classification. Interestingly, differentially expressed proteins (DEPs) were complement cascade C3, C4B and C1S proteins, potentially indicating signs of inflammation (Fig. 1H, Table S2). Prot2 also associates with FBLN2, known to disrupt the basement membrane to promote metastasis in PDAC by the interaction with MUC4 [48]. Likewise, we identified fibrillar collagen COL3A1, one of the EMT‐promoting proteins in PDAC [49], as well as POSTN, a potential myofibroblastic CAF marker found in dense PDAC stroma and particularly linked to ECM‐receptor interaction and PI3K/AKT signaling [50], and matricellular protein THBS1, increased during PDAC progression [51]. In line with their stromal content, Prot2 tumors were particularly enriched for both iCAF and myCAF markers (Fig. S6c).

Both Prot1 and Prot3 were characterized by high tumor cellularity (Fig. 1A–D), but featured distinct biological profiles. Prot1 showed a lipogenic‐like profile with mitochondrial respiration (OXPHOS), fatty acid metabolism and adipogenesis, and with elevated oxidative stress levels compared to Prot2 and Prot3. For instance, we found an increased expression in Prot1 of electron transport chain member ATP5F1 and mitochondrial enzyme ALDH1B1 (Fig. 1G). This lipogenic‐like profile is known to be associated with the epithelial subtype, typically slow proliferative and responsive to chemotherapeutics [52, 53]. Contractile proteins MYH11, ACTG2 and DES were all enriched in Prot1. Interestingly, DES is a marker for pancreatic stellate cells and MYH11 is a marker for a type of CAF similar to stellate cells [54], which could be a stromal signal in this subtype. RNASE3, a homolog of a released toxic granule protein by active eosinophils, was also increased in Prot1. Indeed, some tumors in this subtype were most enriched for the eosinophil signature compared to the other protein groups (Fig. S6c). In relation to Dijk subtypes [28], most of the epithelial (3/4) and half of the secretory (4/8) tumors were gathered in Prot1 group, suggesting this subtype may represent highly‐epithelial/classical PDAC tumors.

Prot3 was particularly inflammatory‐like, enriched for apoptosis, TGFβ signaling, IFN‐gamma response and to a lower extent IFN‐alpha response. TGFβ is known to promote PDAC progression by EMT, invasion and treatment resistance [55]. Depending on *SMAD4* mutation status, TGFβ can signal to active SMAD proteins and promote cell cycle arrest, or drive tumorigenesis when SMADs are inactive [56]. Protein quantification of SMAD4 was however insufficient to find any correlation with *SMAD4* mutation status in the proteomic groups. TGFβ can also contribute to CAF heterogeneity, shifting CAF phenotypes from inflammatory CAFs (iCAF) to myCAFs to, for instance, synthesize ECM proteins and promote EMT. Besides hallmark signatures, we observed differential expression of MIF in Prot3, which could potentially be related to IFN‐gamma and tumor‐associated macrophage activation, leading to an immune‐suppressive environment. Prot3 was enriched for Dijk et al. compound and mesenchymal tumors. Overall, we identified two tumor‐intrinsic subtypes (Prot1 and Prot3), one with epithelial‐like and other with mesenchymal‐like features, and a more stromal and mesenchymal‐like Prot2 subtype. Of note, enriched biology of these protein subtypes strongly aligned with the enriched signatures of three PDAC groups found in a 217 tumor cohort characterized by proteomics [22]. Besides, we observed a reduced survival for the Prot3 patient group (Fig. S7b), possibly due to the immune‐suppressive environment.

### 3.3 Kinase activity profiles challenge single‐hit dependence in PDAC: implications for combination therapies

**Fig. 2:**
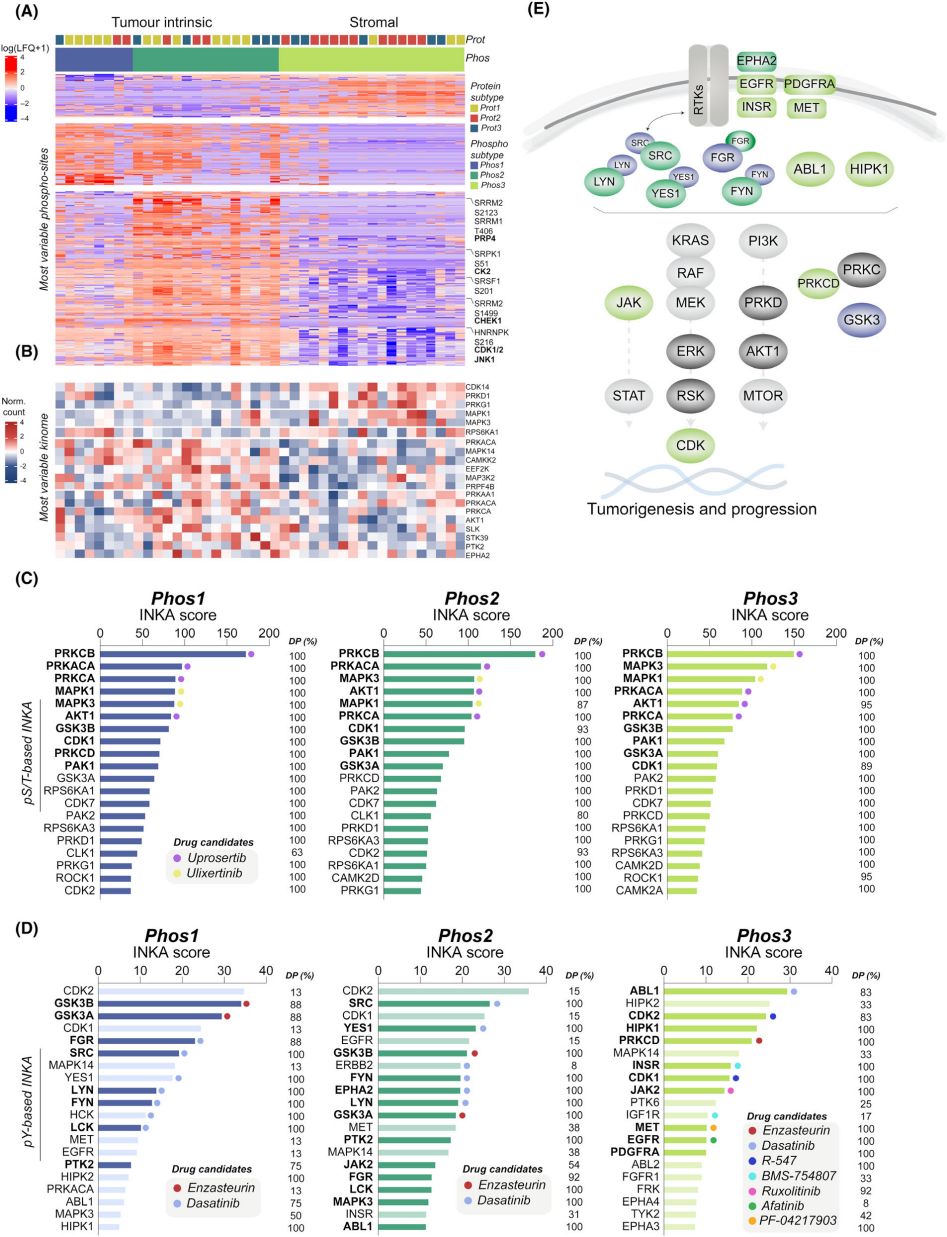
Phosphoproteome analysis of PDAC tumor tissues. (A) Hierarchical clustering of label free quantitation (LFQ) intensities of differentially phosphorylated serine and threonine (pS/T) sites between phosphoproteome subtypes (three group comparison, *n* = 973, FDR ≤ 0.05, Table S3), obtained by consensus clustering (see Fig. S5). (B) Phospho‐kinase abundance of most variable kinome subset (top 20%) based on pS/T phosphoproteomics. (C, D) Kinase activity profiles based on Integrative iNferred Kinase Activity (INKA) scores in Phos1‐3 at (C) pS/T level (Table S4) and (D) phosphotyrosine (pY) level (Table S5). Significantly different pY‐kinase activities between subtypes (using Mann–Whitney test, see Fig. S8 for *P* values) are annotated by a darker color and a bold font. DP across tumor samples is annotated in percentage. Drug candidates were selected based on drug‐kinase relationships and target inhibition efficacy [118]. (E) Mapped kinase signaling from pS/T and pY INKA profiles enriched in Phos1‐3 pancreatic ductal adenocarcinoma (PDAC) subtypes (only significant from Fig. S8 were annotated).

We then examined the global phosphoproteome of all 42 resected PDAC tumors and explored whether PDAC phosphoproteome subtypes uncover novel information not captured at the proteome level. Applying a 10% DP filter to remove noise, we used the top 10% most variable phosphorylations (1496 pS/T sites) as input for consensus clustering. Recent studies have described different phosphoproteomic features in clinical subgroups, which were based on proteomics or multiple omics [22, 23]. This analysis identified three main phosphoproteomics‐based subgroups (Phos1‐3; Fig. 2A, Fig. S5b), the same optimal number as for the proteomic subtypes. Interestingly, by annotating the three protein subtypes, Phos1‐2 showed to be highly tumor‐cell intrinsic, while Phos3 showed to be mostly stroma‐enriched (Fig. 2A). Additionally, by interrogating the most variable pS/T kinome, differential kinase phosphorylation was found in an unsupervised manner between the phospho‐groups (Fig. 2B). Some of the differentially enriched kinases included RPS6KA1 in Phos1, PRKAA1, PRKACA, PRKCA, AKT1, PTK2 and EPHA in Phos2, and PRKD1, PRKG1, MAPK1 and MAPK3 in Phos3. Most variable pY kinome exhibited higher variability among samples (Fig. S6b) with no particular association per subtype.

Subsequently, we investigated differences at the phosphosite level among the phospho‐subtypes and found splicing to be differentially regulated. By group comparison, we identified a total of 973 differentially phosphorylated S/T sites between the three phospho‐groups (Table S3). Protein–protein interaction networks showed high connectivity between phosphorylated proteins per group as expected (Fig. S6a). Phos3 was enriched for 175 phosphorylations occurring in proteins involved in RNA splicing and transcription, the actin cytoskeleton, and cell adhesion (phosphorylation cluster C1). Phos1 and partly Phos2 had significantly more phosphorylations occurring in proteins involved in RNA splicing and axonogenesis (C2). Intriguingly, RNA splicing was enriched in all three phospho‐groups, however differences in phosphorylation events were identified (Table S3b). Phos2 exhibited the highest number of phosphosites associated with splicing (157/196). Notably, there was an enrichment of phosphosites attributed to PRP4, a critical regulator of RNA splicing [57, 58]. Furthermore, in Phos2, we observed an enrichment of HNRNPK, a major pre‐mRNA‐binding protein previously linked to increased tumor growth in KRAS and TP53‐mutated PDAC tumors [59]. HNRNPK was found to be phosphorylated by CDK1/2 and/or JNK1. These upstream kinases, along with TBK1 and ATM, were uniquely identified in Phos2 tumors. Additionally, the splicing regulator SRPK1, whose activation occurs through increased phosphorylation by CK2 kinase, was enriched in these tumors. Interestingly, its expression has been linked to poor outcomes in other cancer types [60]. Remarkably, phosphorylation of the splicing factor and proto‐oncogene SRSF1, known for its association with significant alterations in alternative splicing patterns [61], was uniquely enriched in Phos2. Overall, these findings, coupled with the abundance of phosphosites in splicing‐related proteins, suggest that Phos2 tumors exhibit a highly active cell cycle and RNA splicing machinery. Conversely, Phos3 appeared to have lower levels of splicing, with no enrichment of serine/arginine‐rich splicing factor (SRSF) proteins, compared to Phos1‐2 where multiple SRSFs were identified. Two of these were found to be phosphorylated by AKT1. Interestingly, phosphoprotein abundance of AKT1 was also found to be lower in Phos3 compared to Phos1‐2 (Fig. 2B).

Kinase activity profiling is a valuable tool for exploring therapeutic approaches. To identity hyperactive protein kinases that may differ between phospho‐subtypes, we used the Integrative iNferred Kinase Activity (INKA) pipeline [31] (Tables S4 and S5). Interestingly, kinase activity was particularly high in Phos2 and Phos3 (Fig. S8). By inferring kinase activity, and contrary to the pY kinome, pY‐based INKA profiles did show substantial differences between subtypes. This underscores why integrating both kinase‐ and substrate‐centric information is key to define kinase activity [31, 62]. Compared to Phos2 and Phos3, pY‐based kinase profiles of Phos1 patients showed significantly higher kinase activities of GSK3α and GSK3β, known to be involved in PDAC pathogenesis and progression [63], and FGR (group based INKA profiles, Fig. 2C,D). Based on the global phosphoproteome profiles, Phos2 patients also showed elevated activity of AKT kinases, BRAF, CAMK2, CDKs, MTOR, PRK kinases, RAF1, and SRC (Fig. 2C, Fig. S8b), although kinase ranking remained consistent for all subtypes. Compared to pS/T, pY‐based kinase profiles exhibited greater differences among the three subgroups. Phos2 patient pY profiles displayed higher activity of EPHA2, FYN, LCK, LYN, MAPK3, PTK2, SRC, and YES1 (Fig. 2D, Fig. S8b). Interestingly, stroma‐enriched Phos3 patient profiles displayed significantly higher activity of pY‐based kinases such as EGFR, INSR, MET, PDGFRA, ABL1, ABL2, CDK1, CDK2, HIPK1, JAK2, MAPK1, and PRKCD (Fig. 2D, Fig. S8c). In terms of therapeutic options, pS/T INKA profiles suggest PI3K/AKT and/or MAPK signaling as two main downstream pathways to be targeted regardless subtype. Based on pY INKA profiles, a limited number of drugs should suffice for Phos1 and Phos2 subtypes, i.e., enzasteurin and/or dasatinib for the majority of patients, whereas for stromal Phos3 subtype, drug targets were more diverse and may be selected in an individual profile‐guided manner. In line with this, kinase activities such as CDK1/2, EGFR, ERBB2, or HIPK2 were found high in a small group of patients (DP 8–33%), which underscores the importance of a personalized treatment strategy. Significant kinase activities were then mapped (Fig. 2E), further displaying main signaling differences per subtype.

**Fig. 3:**
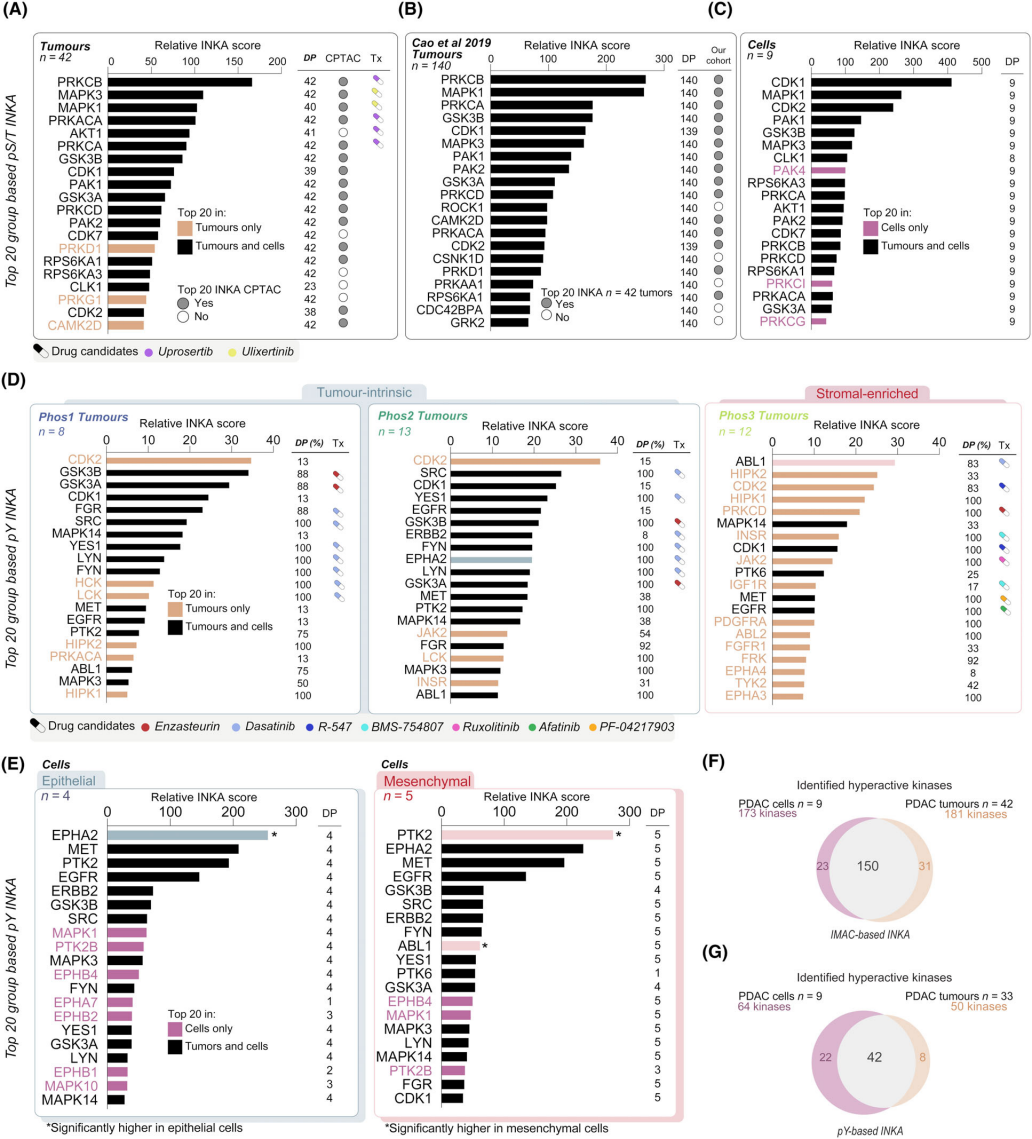
Overlapping kinase activity profiling in SPACIOUS and CPTAC tumor cohorts and PDAC cell lines. (A) Phosphorylated serine and threonine (pS/T) Integrative iNferred Kinase Activity (INKA) group‐based profiles of pancreatic ductal adenocarcinoma (PDAC) tumors of SPACIOUS cohort (*n* = 42), (B) of CPTAC cohort [23] (*n* = 140) and (C) of a PDAC cell panel (*n* = 9) [26]. Top 20 INKA kinase presence in tumors and cells is annotated by orange and magenta colors, respectively. Drug candidates are annotated in relation to Fig. 2. (D) Phosphotyrosine (pY) INKA group‐based profiles of PDAC tumors of Phos1, Phos2 and Phos3 from the SPACIOUS cohort (*n* = 33). Target candidates vary per Phos subtype related to Fig. 2 and Fig. S8. (E) pY INKA group‐based profiles of a PDAC cell panel (*n* = 9), containing four epithelial cell lines and five mesenchymal cell lines. Significance of differential kinase activities (EPHA2 *P* = 0.0124, PTK2 *P* = 0.049) by Mann–Whitney test was annotated with an asterisk (*) from previous study [26]. Kinases EPHA2 and ABL1, associated to tumor intrinsic and to stromal subtypes, were annotated in green and salmon shade, respectively. (F, G) Overlapping INKA kinases between PDAC cell lines and tumors at (F) pS/T level and (G) pY level.

In our analysis, the INKA top 20 ranking of tumor group‐based kinase activities not only confirmed known roles in tumor progression (Fig. 3A,D) but also revealed additional insights through the examination of external datasets and our own preclinical study. By using the INKA pipeline on an external PDAC global phosphoproteomics dataset [23], not only we confirmed 4/5 described kinases in the PDAC phosphoproteomics dataset of Cao et al. (Top 20: CDK7, PAK1, PAK2; hyperactive: SRC), but also revealed additional pS/T activities overlapping with our cohort (Fig. 3A,B), as well as with our own recent preclinical PDAC study [26] (pS/T level in Fig. 3C,F; pY level in Fig. 3G). Notably, the only kinase that was not found in the INKA ranking of the CPTAC cohort was AKT1, that was lost in the required TMT batch‐to‐batch normalization. Overall, these overlaps reinforce our approach and validate our preclinical findings. It is noteworthy that stromal Phos3 tumors exhibited a higher abundance of top 20 tumor‐unique INKA kinases, and is explained by the lack of stroma in cell culture profiles. Despite this, matching epithelial and mesenchymal subtype kinase activities were found between tumor and cell profiles, e.g. mesenchymal cells displayed significantly higher ABL1 kinase activity, in line with the stromal Phos3 profiles (Fig. 3D,E, Fig. S8c). The same was observed for EPHA2 kinase activity, found to be significantly increased both in epithelial cells and tumor‐intrinsic Phos2 subtypes (Fig. 3D,E, Fig. S8b).

Overall, the use of pY‐based profiles provided an additional and actionable layer of information that can be used not only to complement the existing characterization of the PDAC phosphoproteome but to underscore the need for personalized treatment strategies. Furthermore, these kinase activity profiles indicate the need for combination strategies in PDAC.

### 3.4 Comprehensive profiling of mutated genes and phosphoproteomic signatures in PDAC

We sequenced a panel of 31 commonly mutated genes such as *KRAS* (frequency of mutated samples, 95.2%), *TP53* (73.8%), *SMAD4* (21.4%), *CDKN2A* (7.1%), and others (Table S6). Multiple *KRAS* variants were identified at the anticipated frequencies such as G12V (40%), G12D (35%), G12R (10%), G12R/G12V (2.5%), G13D (2.5%), and Q61H (2.5%) [22, 64]. Wildtype *KRAS* was identified in two out of the 42 human tumors, and for three tumors, the allele variant was not available.

**Fig. 4:**
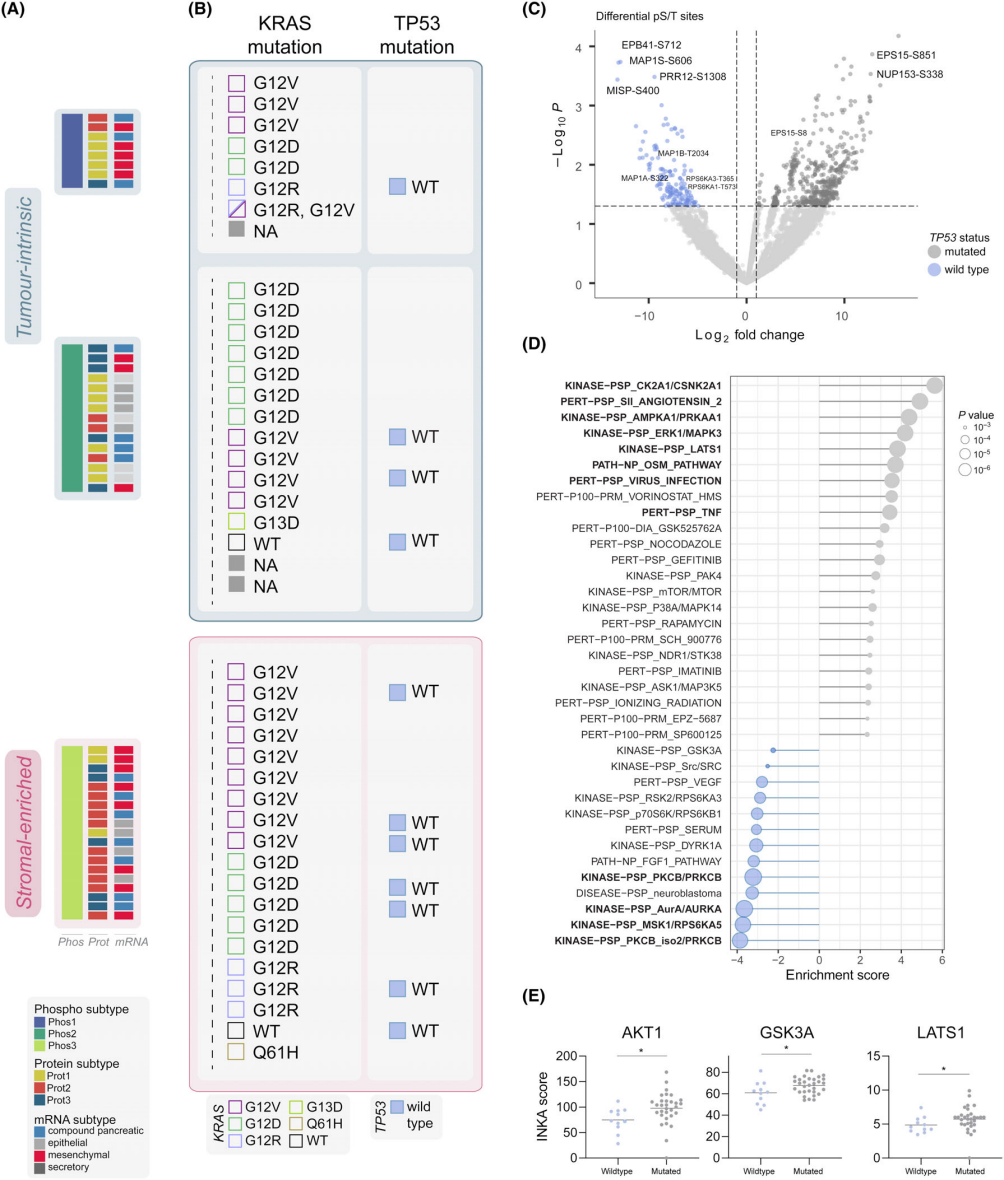
Association of *KRAS* and *TP53* gene mutations with phosphoproteome signatures. (A) Phosphoproteome and proteome subtypes annotated with transcriptomics Dijk et al. subtypes [28]. (B) *KRAS* mutation status and allele variants and *TP53* mutation status (see Table S6). (C) Differentially phosphorylated serine and threonine (pS/T) sites (*n* = 710) in *TP53* mutated (*n* = 31) and *TP53* wild type (*n* = 11) tumors with a fold change cut off of two and *P* ≤ 0.05. Two‐group comparison was performed using unpaired *t*‐test in *limma* statistics. Differential sites were involved in EGFR internalization (i.e., ANKRD13D‐T556, REPS1‐S174, EPS15‐S851), microtubule dynamics and mitotic regulators (i.e., MAP1B‐T2034, MISP‐S400, EPB41‐S712, MAP1S‐S606), cytoskeleton and EMT (FLNA‐S968, VCAN‐T2115) and downstream ERK signaling (RPS6KA3‐T365, RPS6KA1‐T573, NUP153‐S338). See also Table S9. (D) Global post‐translational modification signature enrichment (PTM‐SEA) analysis between *TP53* mutated and *TP53* wild type samples. In bold, significant signatures by adjusted *P* ≤ 0.05. (E) Integrative iNferred Kinase Activity (INKA) of phosphorylated serine and threonine (pS/T) kinases significantly enriched in *TP53* mutated tumors. Significance, annotated with an asterisk, was performed using Mann–Whitney test.

The mutation status within phosphoproteome subtypes revealed distinct patterns, particularly in relation to *KRAS* and *TP53* mutations and associated signaling pathways (Fig. 4A). *KRAS* G12D mutations were mainly observed in Phos2 (7/14 of G12D‐mutated) compared to Phos3 (5/14), whereas G12V mutations were mainly found in Phos3 (9/17 of G12V‐mutated) compared to Phos2 and Phos1 (both 4/17). *KRAS* G12R mutations presented in Phos1 and Phos3, but not in Phos2. Here, *KRAS* dosage at mRNA was slightly higher in Phos2 than in Phos3 (Fig. S6e, *P =* 0.0123). In addition, we identified significantly enriched phosphoproteome signatures associated with the G12D, G12V and G12R *KRAS* alleles (Fig. S9). The G12D allele was associated with the unique enrichment of cell cycle regulators CK2A1 and AMPKA1 kinases, and G12V with the enrichment of CDK1 and SRC kinases, and GLP1 and IL11 pathways. However, cell gene‐dependency data of these proteins did not confirm any unambiguous dependency in G12D or G12V mutated contexts (DepMap data not shown). Overall, these observations confirm the lack of single‐hit dependencies in PDAC, as described in our recent preclinical study [26]. Interestingly, intensity of two phosphosites in MAPK targets (MAPK14‐Y182 and MEF2A‐T108) was enriched in G12D mutated tumors. These results are in line with a recently reported enrichment of Ras/Raf/Mek/Erk cascade [22] and cell cycle [65] in G12D tumors. Differential phosphosites related to this were identified (Fig. S9d and Table S7). It is known that G12D‐mutated tumor cells can signal oncogenic stimuli to stromal cells, the later promoting IGF1R, AXL and AKT signaling and providing a high level of complexity [66]. Overall, our findings support G12D tumors are strongly enriched to cell cycle and RAS signaling and may capture the tumor‐intrinsic compartment, which can be involved in reciprocal signaling with the stromal compartment (Fig. 4B).

In *TP53*‐WT tumors, the *TP53* onco‐suppressive function must be overcome in order to maintain tumor growth. Interestingly, the majority of *TP53* wildtype tumors were classified as Phos3 stromal‐subtype (7/11 of WT *TP53* tumors, Fig. 4B). Notably, mutational status was unaffected by tumor cellularity (Fig. S10e, unpaired *t* test *P = 0.3292*). Thus, we further investigated the phosphoproteomic background of *TP53* mutation status in this PDAC cohort (Fig. 4C–E, Fig. S10, Tables S8 and S9). We identified phosphorylations enriched in wildtype tumors involved in EGFR internalization and EGFR signaling/activation, microtubule dynamics and mitotic regulators, cytoskeleton and EMT, and downstream ERK signaling (Fig. 4C, Fig. S10a). Additionally, Y259 phosphorylation of RASSF1, which regulates TP53 activation [67, 68], was enriched in wildtype tumors (Fig. S10a). By PTM‐SEA analysis, wildtype tumors were found to have significantly higher levels (FDR < 0.05) of PKCB, MSK1/RPS6KA5 and AURKA kinases (Fig. 4D, Fig. S10b). Interestingly, we identified a neuroblastoma signature enriched in *TP53*‐WT tumors (Fig. 4D), which is known to be characterized by non‐mutated *TP53*, further supporting our findings.

In *TP53* mutant tumors, we observed a significant enrichment (FDR < 0.05) of CK2, known to inhibit TP53 tumor suppressor function [69], AMPKA1, known to inhibit autophagy in PDAC and breast cancer [70], ERK1, LATS1, AKT1, and GSK3A kinase signaling and activity (Fig. 4D,E). In addition, we found proteins involved in apoptosis and G2/M arrest enriched in *TP53* mutants (Fig. S10d). By interrogating their function, we observed that in these PDAC tumors, the regulation of apoptosis can serve as a tolerance mechanism towards stress like DNA damage or oxidative stress when TP53 is mutated [71]. This is suggested by the noticeable increase in the expression of SFN (or 14‐3‐3*σ*) observed in TP53‐mutated tumors in both our cohort and the CPTAC cohort (Fig. S10g). The Oncostatin M (OSM) pathway was also enhanced in *TP53* mutated tumors (Fig. 4D); OSM can involve pathways such as ERK1/ERK2, JNK‐p38, and PI3K‐AKT [72], which were also fully or partially enhanced and eventually may regulate *TP53* activity. This ERK‐TP53 association also highlights the crosstalk between the two mutated *KRAS* and *TP53* genes.

### 3.5 Distinct phosphoprotein activities associate with patient outcomes

It has been suggested that *KRAS* dosage rather than allele variants drive tumorigenesis and metastasis [10]. Here, we observed that treatment naïve individuals with an overall survival (OS) lower than 12 months had elevated *KRAS* mRNA levels (Fig. S11c, *P =* 0.0776). KRAS protein levels were too low abundant to confidently verify this change at the protein level (Fig. S11d).

The association between overall survival and the multiple omics (mRNA, protein and phospho) patient groups was investigated by Kaplan–Meier analysis. Due to a small sample size and poor survival in the majority of patients, no significant associations were found among the three omic types. Nonetheless, it was observed that patients classified as Phos1 had the shortest average OS of 13 months (excluding one patient with a survival of 81 months), followed by Phos3 and Phos2 with mean OS durations of 20 and 19 months, respectively (Fig. S7a). Given that a large number of patients received adjuvant chemotherapy and/or palliative treatment (Table S1), which might influence prognosis, we focused on treatment naïve patients (no neo/‐adjuvant and/or palliative treatments received) with low and high survival times (> 29.9 months, based on a bimodal cut‐off, see Fig. S7e,f). Although the number of patients with long (*n* = 3) and short survival (*n* = 20) were not equally distributed, we identified increased signaling of cell cycle kinases CHEK1 and PLK1, PRK, AKT1, and enriched GLP1, NOTCH and KIT receptor pathways in the short survival group (PTM‐SEA, Fig. S11a). Of note, in the long survival group, the enrichment of cell cycle arrest mediator CDC7 was identified.

**Fig. 5:**
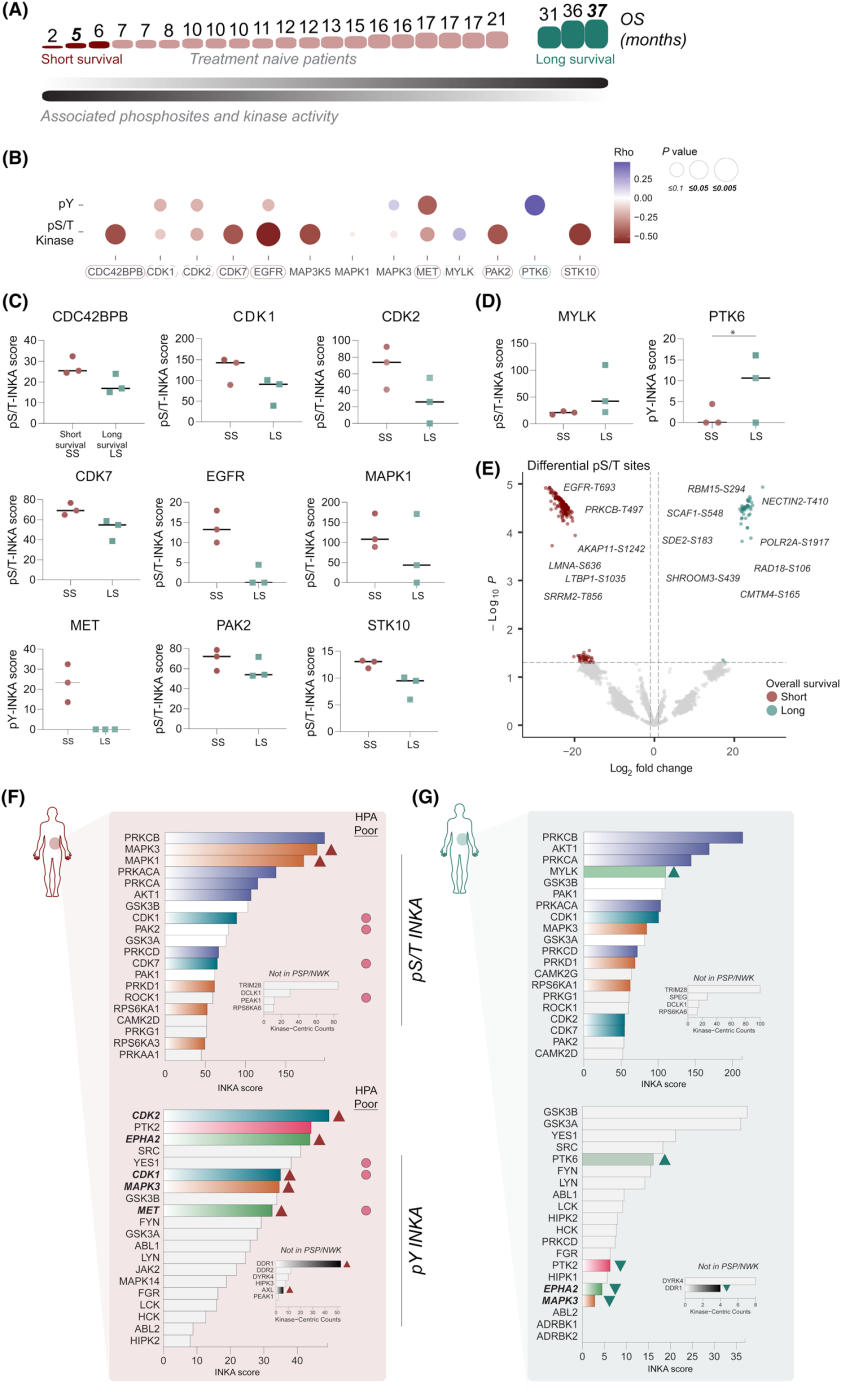
Kinase activity in short and long survival (treatment naïve) patients. (A) Spearman correlation of phosphorylated serine and threonine (pS/T)‐based Integrative iNferred Kinase Activity (INKA) kinases. (B) Overview of Spearman correlation coefficients and *P* values between INKA kinases at phosphotyrosine (pY) and pS/T level and overall survival in treatment naïve patient subset. Boxes suggest kinase relevance in prognosis outcomes. (C) Elevated INKA scores of pS/T and pY‐based kinases in short survival (SS) patients. Short and long survival groups were defined with patients with opposing outcomes. (D) Elevated INKA scores of pS/T and pY‐based kinases in long survival (LS) patients. *P = 0.0406, significance performed using Mann–Whitney test. (E) Volcano plot representing differential pS/T sites between poor and better survival patients (3 vs 3) with fold change two and *P* ≤ 0.05. Two‐group comparison was performed using unpaired *t*‐test in *limma* statistics. See also Table S10. (F) pS/T and pY‐based INKA profile of poor survival patient (overall survival, OS = 5 months). Significant poor prognostic markers by Human Protein Atlas (HPA) were annotated. (G) pS/T and pY‐based INKA profile of long survival patient (OS = 37 months). For both patients, kinase‐centric counts of kinases with no kinase‐substrate relations in PSP/NWK are also displayed. INKA kinases were colored to pinpoint common members of signaling pathways and to highlight the differences between patients.

By using the single‐sample integrative inferred kinase activity (INKA) scoring, we explored hyperactive kinases associated with survival per tumor in the pY phosphoproteome and global (pS/T) phosphoproteome (Fig. 5 and Fig. S11). Kinase activity differed substantially between long and short survival groups despite the small number of treatment naïve long survival patients in our cohort (Fig. S11a,b). Moreover, we identified by spearman correlation several kinases significantly associated with poor overall survival such as receptor tyrosine kinases MET and EGFR (Fig. 5B,C). Other kinases slightly elevated in the poor outcome group from our cohort include: (a) CDC42BPB, a kinase involved in cytoskeleton reorganization and migration; (b) PAK2 kinase, a poor prognostic marker in PDAC [73] and found to be activated by TGFβ secretion mediated by fibroblasts [74] and involved in cytoskeleton reorganization, and (c) cell cycle regulators STK10 and CDK7, both involved in tumor progression [75]. Interestingly, CDK7 inhibition has been recently combined *in vivo* with gemcitabine and nab‐paclitaxel to overcome chemoresistance in PDAC with strong synergistic results [76], and a phase I clinical trial is now on going (clinicaltrials.gov, NCT04247126). Although MAPK1/3 were not significant (Fig. 5B,C), by reanalyzing Cao et al. [23] data on a subset of tumors for which overall survival data was available (*n* = 67), we identified MAPK1 activity to slightly correlate with poor overall survival (ρ = −0.251, *P =* 0.041), together with SRPK2 (ρ = −0.25, *P =* 0.041) and ULK1 (ρ = −0.285, *P =* 0.019) kinases. By correlating the expression of these poor outcome associated kinases to the expression of proliferation marker MKI67 in the larger cohort of Cao et al., we could confirm MET (ρ = 0.22, *P =* 0.007), CDK1 (ρ = 0.63, *P =* 9.12E‐17) and CDK2 (ρ = 0.27, *P =* 5.7E‐6). Overall, these findings confirm that poor outcome PDAC patients exhibit aggressive cancer behavior, marked by robust proliferative signals.

We additionally found PTK6 activity to significantly correlate with higher overall survival rates (Fig. 5D). Additionally, we observed differential phosphorylations at pS/T level and at pY level (Fig. 5E, Fig. S11b, Tables S10 and S11) in the short survival patients (*n* = 3), such as EGFR‐T693, PRKCB‐T497, LMNA‐S636, and LTBP1‐S1035; and RBM15‐S294, NECTIN2‐T410 and SCAF1‐S548 in the long survival group (*n* = 3).

By comparing the (top 20) individual INKA profiles of two patients with opposing survival outcomes (5 vs 37 months), we observed strongly elevated MAPK signaling at pS/T and pY level in the short survival patient (Fig. 5E) as was evident from both the high INKA score and rank, while PI3K‐AKT signaling was high in both profiles. Interestingly, at the pY level, not only CDK1 and CDK2 but also EPHA2, MET, and DDR1 receptor tyrosine kinases were hyperactive in the poor survival patient profile and absent in the long survival profile. Of note, several of these kinases have been defined by Human Protein Atlas (HPA) database as poor prognostic markers for PDAC patients, and others, such as the DDR1 receptor have also been suggested to predict poor outcomes [77, 78, 79]. Interestingly, the long survival patient showed a 5‐fold increased MYLK kinase hyperactivity compared to the poor survival patient. This difference in MYLK activity was also observed between the extreme outcome groups (Fig. 5D). This kinase is commonly expressed in epithelial cells and its downregulation has been associated with an increase in motility and invasiveness in several cancer types [80, 81, 82]. Interestingly, MYLK expression significantly and inversely correlated with increased expression of cycle kinases CDK1 and CDK2 and proliferative marker MKI67 in the larger PDAC cohort of Cao et al. (Fig. S11e). Likewise, in this external cohort, MYLK expression was found to be increased in patients with improved outcomes (Fig. S11f,g), which further supports the significance of this kinase as a potential indicator for better prognosis. Besides, its loss has been associated with elevated EGFR and ERK signaling, which was observed in the short survival group with an additional increase of CDK signaling (Fig. 5B).

Our focus on patients with extreme outcomes allowed us to identify specific kinases that likely play a direct role in tumor progression. These kinases hold potential as valuable prognostic markers for PDAC.

## 4 Discussion

Quantitative profiling of tumors following a phosphoproteomic approach has shown to generate a precise and comprehensive data set with thousands of phosphosites and proteins. It has led to the discovery of clinically relevant markers, for diagnosis and prognostic purposes, and has unraveled druggable targets in multiple cancer types [16, 20, 32, 83, 84, 85]. So far, only proteogenomic efforts, especially those including phosphoproteomics, have been able to come up with therapeutic strategies in multiple cancer types [16, 19, 22, 86, 87, 88].

In this study, we report a comprehensive phosphoproteome and proteome of PDAC tissues from which three main subtypes were derived. Despite using a subset (42/90) of Dijk et al. [28] cohort tumors, different RNA subgroups aligned with the three Phos subtypes. In line with this, all the epithelial RNA classified tumors were captured in the Phos2 subtype, Phos1 subtype consisted only of mesenchymal and compound tumors, and stroma‐enriched Phos3 showed a mixed profile of mesenchymal, compound and secretory tumors.

The tumor and stromal cell enrichment was largely reflected in the proteome‐based subtypes. We identified two tumor‐intrinsic subtypes, Prot1, and Prot3, the latter one potentially being an intermediate state to a more stromal and basal‐like subtype, which was captured by the Prot2 subtype. Most of the enriched TME signatures were myCAF and iCAF, mostly in Prot1 and Prot2 clusters. The MAPK and JAK–STAT pathways are critically involved in the activation of these cells, playing a role in promoting tumorigenesis and cancer invasion [89]. Furthermore, TGFβ secretion is known to be also mediated by CAFs, stimulating PDAC growth and promoting EMT [89, 90, 91]. Interestingly, TGFβ signaling was the highest in the Prot3 cluster, suggesting Prot3 may recapitulate this interplay tumor‐TME cells to activate different oncogenic pathways [92]. Despite not including normal adjacent tissues, we identified the PDAC biology found in tumor vs normal adjacent tissue comparisons performed in two recent studies, such as extracellular matrix organization, actin filament organization, complement and coagulation, focal adhesion, fatty acid metabolism and platelet activation [22, 23]. In Cao et al., two main subtypes capturing classical and basal‐like features were identified by integrating multiple omic layers and leaving out low cellularity samples [23], and in Tong et al., protein expression clustering divided the cohort into three main proteomic subtypes, with differences in survival, enriched biology and kinase activity, mainly based on phosphosubstrate abundance. Something similar was inferred from the PDAC proteogenomic study by Hyeon et al. [24], whereby a first‐step clustering tumors were classified into three main subtypes by using RNA, protein or phosphoprotein data. To the best of our knowledge, none of these cohorts were classified into subtypes by using phosphoproteomic features alone. Unfortunately, the dataset by Tong et al. as well as the article and dataset by Hyeon et al., were not accessible for download. This underscores the pivotal importance of data accessibility to enable the reuse of data in accordance with FAIR principles for biomedical research [93].

Kinases play a key role in regulating alternative splicing events [60], which hold significant physiological importance in conferring functional diversity within tumors, including PDAC [59, 61]. In fact, this complex mechanism is known to regulate the post‐transcriptional gene expression during, for example, tumor progression and treatment resistance in PDAC [94, 95]. At the phosphoproteomic level, we identified members of RNA splicing and spliceosome such as SRRM1/2, TRA2A/B or SRSF proteins, which were different across the Phos subtypes. Interestingly, a recent re‐analysis of a cohort of *n* = 177 PDAC tumors also found three patient subtypes based on alternative splicing data [96]. These differences in splicing‐related phosphorylation events provide valuable insights into comprehending the diversity within these distinct phosphoproteomic PDAC groups.

Pancreatic ductal adenocarcinoma is characterized by the lack of druggable drivers, e.g. KRAS, although emerging studies are showing promising results by more selective KRAS inhibitors [97]. Our study aimed to identify drug‐therapeutic strategies for PDAC tumors, which may be used as well to prevent signaling feedback loops in future KRAS targeting approaches. Accordingly, INKA analysis nominated kinase activities differentially enriched in the three subtypes that may be relevant at the prognostic level and may be considered as druggable targets in PDAC. We observed that kinase activity profiles varied between tumor intrinsic subtypes, with a highly hyperactive Phos2 (hyperactivity of PI3K/AKT and mTOR signaling members, PTK2, SRC, EPHA2 kinases) and a less active Phos1 subtype. Furthermore, the activation of AKT2 and PRKACB kinases in Prot3 subtype (distributed in Phos1 and Phos2) was found in the previously identified S‐III subtype by Tong et al. [22]. The stromal‐like subtype, Phos3, displayed significantly higher levels of HIPK, EGFR, MET, PDGFRa, INSR, JAK2, ABL1/2, and PRKCD. Recently, PRKCD and HIPK2 have also been identified high in PDAC vs NATs, with PRKCD as a poor prognosis marker by Tong et al. [22], however we did not observe an association with survival. Moreover, activated INSR/IGF1R and JAK kinases in Phos3 could be associated with EMT and cancer stemness [98], as well as the immunosuppression [99]. HIPK1 in this subtype could be involved in CAF differentiation and invasion [100, 101]. Altogether, Phos3 recapitulated kinase activities that may play a role in the interaction with the tumor microenvironment and may serve to design better therapeutic regimes and tackle the stromal barrier. Patient group profiles underscored not only the need for combination treatments but also for personalized treatment strategies. PI3K/AKT and MAPK pathways showed the highest hyperactivity in the pS/T‐based INKA ranking regardless subtype. This supports recent preclinical findings of a combination screening study and of our phosphoproteomic guided study, where low‐dose drug combinations containing PI3K/AKT and MAPK inhibitors showed higher effectivity and synergism [26, 102]. Heterogeneity in signaling was found at the pY‐based INKA level between patients uncovering different drivers in subsets of patient samples, especially among the stroma‐enriched Phos3 group, which underscores the heterogeneity of the stroma and highlights the need for a more sophisticated and personalized approach.

Interestingly, EPHA2, the expression of which has been associated with the immunosuppressive environment in PDAC [103], turned out to be the most active receptor tyrosine kinase. Others, such as MET, INSR, EGFR and FGFR1 were found to be particularly high in tumors enriched in stroma, underscoring the importance of individual profiles in guiding therapy selection. Of note, we previously reported EPHA2 and EGFR to be elevated in the tumor cell compartment of PDAC tissues [46]. Our reanalysis and inspection of the phosphoproteomics data of two recent proteogenomic studies with larger PDAC cohorts that also included normal tissues [22, 23] underscores the importance of the kinase activities in our cohort and their cancer‐association. This supports the clinical utility and relevance of our kinase activity profiling in the absence of normal adjacent tissues.

Differences in *KRAS* allele dosing were further investigated in this cohort. Enrichment of cell cycle regulators and MAPK targets was found in G12D mutated tumors, in line with Tong et al. [22]. The most commonly mutated gene in human tumors is *TP53* [104]. Its transcriptional activity is mainly regulated by acetylation and phosphorylation [105]. Interestingly, N‐terminal TP53 phosphorylation activity (by CK1 and ERK), was found to be enriched in *TP53*‐mutant PDAC tumors, whereas most of the C‐terminal phosphorylations (by AURKA, PKC) were enriched in *TP53*‐WT PDAC tumors. We additionally observed elevated PKCB kinase activity in *TP53*‐WT tumors, which could be attributed to the role of PKC in regulating the tumor suppressor activity of *TP53* by stabilizing the wildtype protein [106, 107]. On the other hand, mutated‐*TP53* tumors exhibited higher AMPK and LATS1 activities, and a higher glycolytic and proliferative profile, shown here by higher TCA and cell cycle. AMPK kinase is known to upregulate glycolysis, inhibit mTOR [108], and to regulate autophagy and lysosomal scavenging in PDAC, which can promote immune‐evasion [109]. Interestingly, hyperactive KRAS signaling is able to promote LATS1/MDM2/*TP53* apoptotic signaling [110, 111, 112] which again supports the crosstalk between *KRAS* and *TP53* gene functions to promote PDAC invasion and growth. Despite not identifying the previously reported phosphorylation sites affected by *TP53* mutated status [23] in this cohort, other residues upon same proteins were identified, with different mutated and wildtype associations (Fig. S10f).

Potential prognostic markers were elucidated in the kinase profiles of extreme outcome patients. The majority of our cohort was classified as short survival tumors, and were treated with different regimes, which added complexity to the analysis. Short survival tumors displayed higher kinase activity of MET, EGFR, STK10, CDC42BPB, CDK7, and PAK2, versus PTK6 and MYLK, which were higher in long survival tumors. Interestingly, in PDAC, elevated PTK6 levels are shown to attenuate tumor growth *in vivo* after gemcitabine treatment *in vivo* [113]. Notably, MYLK association with better outcomes was also observed in a larger PDAC cohort, further supporting our approach.

## 5 Conclusions

Despite the rapid development of phosphoproteomics as a valuable functional platform for the discovery of new treatments, its integration into routine clinical practice is still pending [20, 26, 114, 115]. Our INKA analysis approach, which can rank kinase activities in individual tumors, is optimally suited to specifically prioritize actionable kinases with targeting purposes [20, 21, 116]. This phosphoproteomic study contributes to better define PDAC tumors by dissecting their biology with functionally relevant data and analysis tools and suggests potential therapeutic options for the different phosphoproteomic subtypes to eventually improve patient outcomes in the near future.

## Conflict of interest

MFB has received research funding from Celgene, Frame Therapeutics, and Lead Pharma. He has acted as a consultant to Servier. None of these were involved in the design of the study or the drafting of this manuscript.

## Author contributions

AV‐M collected tumor samples, designed, analyzed the data, performed bioinformatic analyses, and wrote the manuscript. FD and HH contributed to sample collection. JV assessed pathology and tumor cellularity. RRG‐H performed the phosphoproteomic sample‐processing. SRP performed the mass spectrometry measurements and analyzed the data. TVP, SRP, JCK, AAH, and AV‐M performed the bioinformatic analyses. MFB, CRJ, and EG designed and supervised the study and wrote the manuscript.

### Peer review

The peer review history for this article is available at https://www.webofscience.com/api/gateway/wos/peer‐review/10.1002/1878‐0261.13625.

## Supporting information


**Fig. S1.** Large‐scale proteomic and phosphoproteomic analysis of pancreatic ductal adenocarcinoma.
**Fig. S2.** Data overview of the pS/T phosphoproteome of pancreatic ductal adenocarcinoma.
**Fig. S3.** Data overview of the pY phosphoproteome of pancreatic ductal adenocarcinoma.
**Fig. S4.** Data overview of the proteome of pancreatic ductal adenocarcinoma.
**Fig. S5.** Consensus clustering analyses of proteome and phosphoproteome.
**Fig. S6.** Comprehensive analysis of PDAC (phospho)proteome subtypes.
**Fig. S7.** Overall survival analysis of subtypes based on mRNA, proteome and phosphoproteome.
**Fig. S8.** Differential kinase activities among the identified PDAC phosphoproteome subtypes.
**Fig. S9.** Phosphoproteomic signatures among *KRAS* alleles G12D, G12V and G12R.
**Fig. S10.** Association of TP53 gene mutations with pY phosphoproteome.
**Fig. S11.** Phosphoproteome differences in short and long survival (treatment naïve) patients.
**Table S1.** Clinicopathological characteristics of the subset (42/90) of SPACIOUS cohort tumors.
**Table S2.** Differential proteins between the proteome subtypes.
**Table S3.** Differential pS/T phosphosites between the phosphoproteome subtypes.
**Table S4.** pS/T‐based INKA profiling of 42 PDAC tumors.
**Table S5.** pY‐based INKA profiling of 33 PDAC tumors.
**Table S6.** Mutation sequencing panel of frequently mutated genes in 42 PDAC tumors.
**Table S7.** Differential analysis of pS/T sites between *KRAS* G12D mutated and the rest of tumors (G12V, G12R).
**Table S8.** Differential analysis of pS/T sites between *TP53* mutated and wildtype tumors.
**Table S9.** Differential analysis of pY sites between *TP53* mutated and wildtype tumors.
**Table S10.** Differential analysis of pS/T sites between long (*n* = 3) and short survival (*n* = 3) patients.
**Table S11.** Differential analysis of pY sites between long (*n* = 3) and short survival (*n* = 3) patients.

## Data Availability

The mass spectrometry proteomics data have been deposited to the ProteomeXchange Consortium via the PRIDE [117] partner repository with the dataset identifier PXD043111. Human Swiss‐Prot database used for raw data search was acquired from the UniProt database (https://www.uniprot.org/).
