# Supplementary material for: Kinase activities in pancreatic ductal adenocarcinoma with prognostic and therapeutic avenues
Source: Mol Oncol. 2024 Apr 22;18(8):2020–41. doi: 10.1002/1878-0261.13625 (PMC11306541; doi:10.1002/1878-0261.13625)
Supplement: Supplementary file 1 — Fig. S1. Large‐scale proteomic and phosphoproteomic analysis of pancreatic ductal adenocarcinoma. Fig. S2. Data overview of the pS/T phosphoproteome of pancreatic ductal adenocarcinoma. Fig. S3. Data overview of the pY phosphoproteome of pancreatic ductal adenocarcinoma. Fig. S4. Data overview of the proteome of pancreatic ductal adenocarcinoma. Fig. S5. Consensus clustering analyses of proteome and phosphoproteome. Fig. S6. Comprehensive analysis of PDAC (phospho)proteome subtypes. Fig. S7. Overall survival analysis of subtypes based on mRNA, proteome and phosphoproteome. Fig. S8. Differential kinase activities among the identified PDAC phosphoproteome subtypes. Fig. S9. Phosphoproteomic signatures among KRAS alleles G12D, G12V and G12R. Fig. S10. Association of TP53 gene mutations with pY phosphoproteome. Fig. S11. Phosphoproteome differences in short and long survival (treatment naïve) patients. Table S1. Clinicopathological characteristics of the subset (42/90) of SPACIOUS cohort tumors. Table S2. Differential proteins between the proteome subtypes. Table S3. Differential pS/T phosphosites between the phosphoproteome subtypes. Table S4. pS/T‐based INKA profiling of 42 PDAC tumors. Table S5. pY‐based INKA profiling of 33 PDAC tumors. Table S6. Mutation sequencing panel of frequently mutated genes in 42 PDAC tumors. Table S7. Differential analysis of pS/T sites between KRAS G12D mutated and the rest of tumors (G12V, G12R). Table S8. Differential analysis of pS/T sites between TP53 mutated and wildtype tumors. Table S9. Differential analysis of pY sites between TP53 mutated and wildtype tumors. Table S10. Differential analysis of pS/T sites between long (n = 3) and short survival (n = 3) patients. Table S11. Differential analysis of pY sites between long (n = 3) and short survival (n = 3) patients. [file MOL2-18-2020-s001.zip › mol213625-sup-0022-Legends.docx]

**SUPPLEMENTARY LEGENDS**

**Supplementary Table 1.** Clinicopathological characteristics of the subset (42/90) of SPACIOUS cohort tumours.

**Supplementary Table 2.** Differential proteins between the proteome subtypes.

**Supplementary Table 3.** Differential pS/T phosphosites between the phosphoproteome subtypes.**a.** Significantly differential pS/T sites between the phosphoproteome subtypes (FDR≤0.05). **b.** Distinct pS/T sites upon splicing members among phospho-subtypes.

**Supplementary Table 4.** pS/T-based INKA profiling of 42 PDAC tumours.

**Supplementary Table 5.** pY-based INKA profiling of 33 PDAC tumours.

**Supplementary Table 6.** Mutation sequencing panel of frequently mutated genes in 42 PDAC tumours.

**Supplementary Table 7.** Differential analysis of pS/T sites between *KRAS* G12D mutated and the rest of tumours (G12V, G12R).

**Supplementary Table 8.** Differential analysis of pS/T sites between *TP53* mutated and wildtype tumours.

**Supplementary Table 9.** Differential analysis of pY sites between *TP53* mutated and wildtype tumours.

**Supplementary Table 10.** Differential analysis of pS/T sites between long (n=3) and short survival (n=3) patients.

**Supplementary Table 11.** Differential analysis of pY sites between long (n=3) and short survival (n=3) patients.

**Supplementary Figure 1. Large-scale proteomic and phosphoproteomic analysis of pancreatic ductal adenocarcinoma. a.** Study workflow to illustrate optimum cutting temperature (OCT) removal and the subsequent phosphoproteome profiling of forty two PDAC samples. **b-d.** Unsupervised hierarchical clustering heatmap of **b.** n=2122 pY sites in 501 phosphoproteins, **c.** 14693 pS/T sites in 5412 phosphoproteins, and **d.** 5857 proteins identified in PDAC tissues and PDAC pooled cell and internal control cell replicates.

**Supplementary Figure 2. Data overview of the pS/T phosphoproteome of pancreatic ductal adenocarcinoma a.** Serine (S) and threonine (T) phosphosite identifications per tumour sample. **b.** Identifications of two sample replicates of samples #16 and #53. **c.** Identifications of two sample replicates of PDAC-cell line pools. **d.** Identifications of five sample replicates of internal control cell line HCT116. **e.** Distribution of normalized phosphosite intensities. Blue line pinpoints the LFQ intensity threshold higher than 2. **f.** Spearman correlation between pS/T identifications and tumour cellularity. **g.** Pearson correlation of LFQ intensities of 1264 pS/T identifications by present in all PDAC tumours.

**Supplementary Figure 3. Data overview of the pY phosphoproteome of pancreatic ductal adenocarcinoma. a.** Tyrosine (Y) phosphosite identifications per tumour sample. **b.** Identifications of two sample replicates of samples #16 and #53. **c.** Identifications of two sample replicates of PDAC-cell line pools. **d.** Identifications of six sample replicates of internal control cell line HCT116. **e.** Distribution of normalized phosphosite intensities. Blue line pinpoints the LFQ intensity threshold higher than 2. **f.** Spearman correlation between pY identifications and tumour cellularity. **g**. Pearson correlation of LFQ intensities of 962 pY identifications present in 33 of 42 PDAC samples. **h.** Phosphotyrosine levels were evaluated prior to pY capture by Western blotting. Samples #16 and #53 had two MS-replicates, but were loaded once in the gel, thus lanes are duplicated in this panel.

**Supplementary Figure 4. Data overview of the proteome of pancreatic ductal adenocarcinoma. a.** Protein identifications per tumour sample. **b.** Identifications of two sample replicates of samples #16 and #53. **c.** Identifications of two sample replicates of PDAC-cell line pools. **d.** Identifications of six sample replicates of internal control cell line HCT116. **e.** Distribution of normalized protein counts. Blue line pinpoints the Normalized counts higher than 1.5. **f.** Spearman correlation between protein identifications and tumour cellularity. **g.** Pearson correlation of normalized protein counts present in all PDAC tumours.

**Supplementary Figure 5. Consensus clustering analyses of proteome and phosphoproteome.** Consensus clustering was performed on the **a.** protein expression data set (n=370 proteins) and **b.** pS/T phosphoproteomic data set (n=1496 phosphosites) after a data presence filtering of 10% and top 10% most variable by median absolute deviation. For both data types, the optimal cluster number was found to be three. Alternative filtering options also generated three clusters.

**Supplementary Figure 6. Comprehensive analysis of PDAC (phospho)proteome subtypes. a.** Protein-protein interaction network of differentially phosphorylated proteins and biology functions per phospho-subtype. C1 cluster highly phosphorylated in Phos3, C2 cluster mainly in Phos1, and C3 cluster in Phos2. **b.** Phospho-kinase abundance of most variable kinome (top 20%, n=15) based on pY phosphoproteomics. **c-d.** ssGSEA averaged-enrichment scores of immune signatures(120–122) between **c.** proteome subtypes and **d.** phosphoproteome subtypes. * significant differences (*P* ≤ 0.05, unpaired t test). **e-f.** KRAS dosage based on **e.** mRNA levels (*P=* 0.0123, unpaired t test) and **f.** protein levels.

**Supplementary Figure 7. Overall survival analysis. a-d.** Kaplan Meier analysis of subtypes obtained by **a.** global phosphoproteomics, **b.** proteomics, **c.** mRNA PurIST(123) and **d.** mRNA Dijk et al., 2020(28). **e-f.** Bimodal cut off of overall survival of **e.** treatment naïve patients (n=23 samples) and **f.** all patients (n=42 samples).

**Supplementary Figure 8. Differential kinase activities among the identified PDAC phosphoproteome subtypes. a-c.** Significantly different hyperactivities (pS/T and pY INKA) in **a.** Phos1, **b.** Phos2 and **c.** Phos3 subtypes. Each dot represents the INKA score of a kinase for each patient. Significance is annotated by unpaired t-test. See also Supplementary Tables 4 and 5.

**Supplementary Figure 9. Phosphoproteomic signatures among *KRAS* alleles G12D, G12V and G12R. a-c.** Global PTM-SEA pY-based analysis between **a.** G12D and G12V, **b.** G12V and G12R, and **c.** G12D and G12V. In bold, significant signatures by *adjusted P* < 0.05. **d.** Differentially phosphorylated S/T sites in *KRAS* G12D mutated (n=14) and the rest (G12V and G12R, n=20) tumours with a fold change cut off of two and *P* ≤ 0.05. See Supplementary Table 7. **e.** Summary of enriched pathways in G12D and G12V alleles.

**Supplementary Figure 10. Association of TP53 gene mutations with pY phosphoproteome. a.** Differentially phosphorylated Y sites (n=24) in TP53 mutated (n=27) and TP53 wild type (n=6) tumours with a fold change cut off of two and *P* < 0.05. Differential sites were involved in EGFR signalling/activation (i.e. EGFR-Y1172, PTPN11-Y542, GAB1-Y659/Y627, INPPL1-Y986, EPS8-Y491), cytoskeleton and epithelial-to-mesenchymal transition (NUCB2-Y169, PALLD-Y731) and downstream ERK signalling (DOK2-Y299) (RPS6KA3-T365, RPS6KA1-T573, NUP153-S338). See also Supplementary Table 8. **b.** Global PTM-SEA pY-based analysis between TP53 mutated and TP53 wild type samples. In bold, significant signatures by *adjusted P* ≤ 0.05. **c-d.** Representative gene ontology terms (GO BP, MF, CC and REACTOME) of differentially expressed and phosphorylated proteins in **c.** TP53 wild type tumours and in **d.** TP53 mutated tumours. **e.** Tumour cellularity among TP53-wildtype and mutated tumours (unpaired t test *P =* 0.3292). **f.** Significant enrichment (*P* ≤ 0.05) of phosphosites newly described, additional to phosphosites observed by Cao et al.(23). **g.** Normalized protein counts and protein abundance ratio of SFN between TP53-mutated and wildtype tumours in our cohort (n=42 tumours) and in Cao et al. cohort (n=140 tumours, retrieved from cBioPortal). Statistical significance was calculated with the non-parametric Mann Whitney unpaired t-test.

**Supplementary Figure 11**. **Phosphoproteome differences in short and long survival (treatment naïve) patients. a.** Global PTM-SEA between short (n=20) and long survival (n=3) patients. Normalized enrichment scores of significant signatures (P ≤0.05) are plotted for both groups. **b.** Differential pY-sites of extreme outcome patients (2-6 months vs 31-37 months) with fold change higher than two and *P ≤ 0.05*. See also Supplementary Table 11. **c-d.** KRAS dosage based on **c.** mRNA levels (*P =* 0.0776, unpaired t test), and **d.** protein levels. **e.** Spearman correlations between MYLK expression and CDK, EGFR, MAPK and MKI67 expression. Data from Cao et al. cohort (n=140 tumours, retrieved from cBioPortal). **f-g.** Differences in MYLK **f.** expression and **g.** phosphorylation for clinical outcomes such as residual tumour, tumour stage and vital status. Data from Cao et al. cohort (n=140 tumours, retrieved from cBioPortal). Statistical significance was calculated with the non-parametric Mann Whitney unpaired t-test.
