# Supplementary figures and images for: Kinase activities in pancreatic ductal adenocarcinoma with prognostic and therapeutic avenues
Source: Mol Oncol. 2024 Apr 22;18(8):2020–41. doi: 10.1002/1878-0261.13625 (PMC11306541; doi:10.1002/1878-0261.13625)

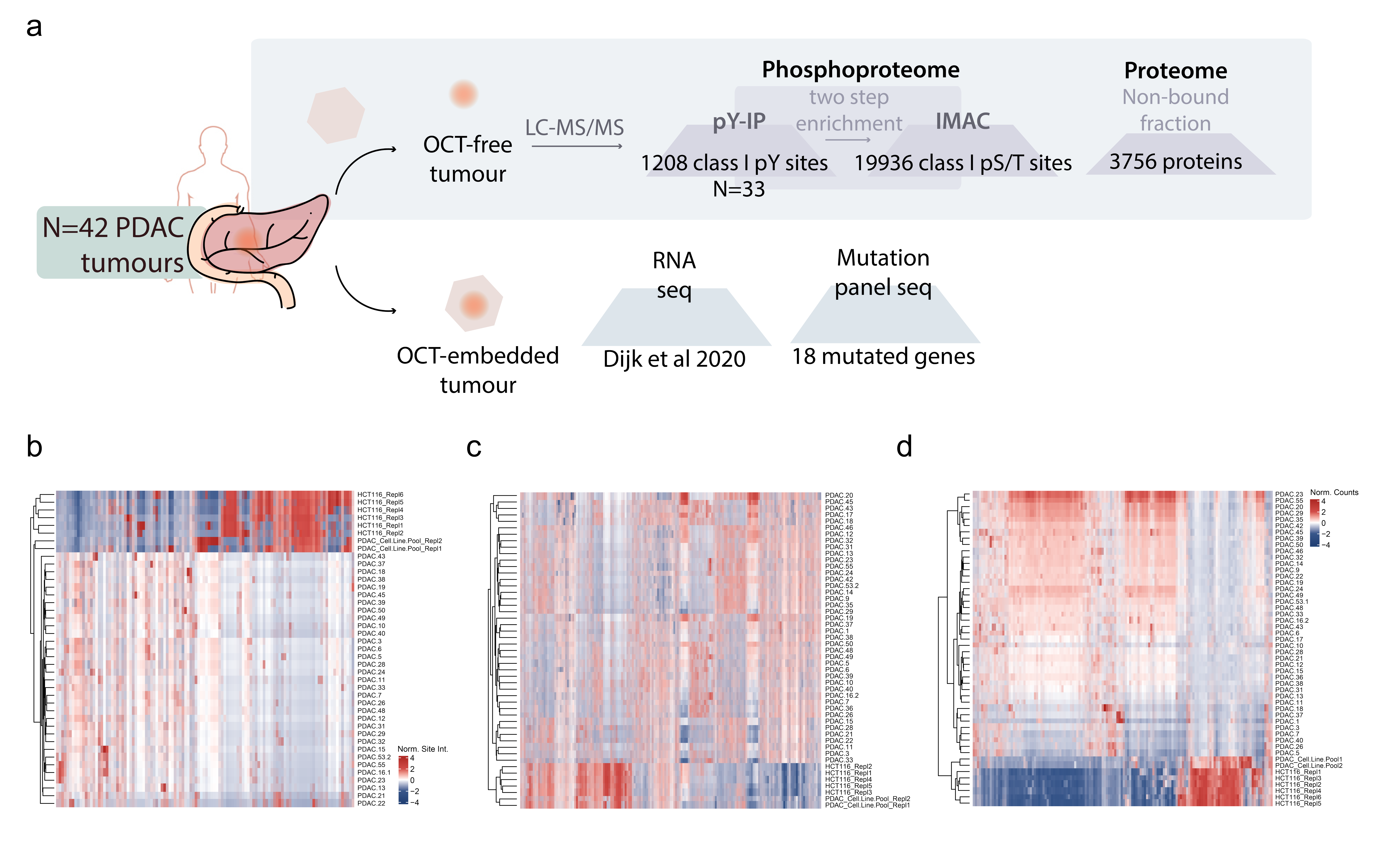

Supplement: Supplementary file 1 — Fig. S1. Large‐scale proteomic and phosphoproteomic analysis of pancreatic ductal adenocarcinoma. Fig. S2. Data overview of the pS/T phosphoproteome of pancreatic ductal adenocarcinoma. Fig. S3. Data overview of the pY phosphoproteome of pancreatic ductal adenocarcinoma. Fig. S4. Data overview of the proteome of pancreatic ductal adenocarcinoma. Fig. S5. Consensus clustering analyses of proteome and phosphoproteome. Fig. S6. Comprehensive analysis of PDAC (phospho)proteome subtypes. Fig. S7. Overall survival analysis of subtypes based on mRNA, proteome and phosphoproteome. Fig. S8. Differential kinase activities among the identified PDAC phosphoproteome subtypes. Fig. S9. Phosphoproteomic signatures among KRAS alleles G12D, G12V and G12R. Fig. S10. Association of TP53 gene mutations with pY phosphoproteome. Fig. S11. Phosphoproteome differences in short and long survival (treatment naïve) patients. Table S1. Clinicopathological characteristics of the subset (42/90) of SPACIOUS cohort tumors. Table S2. Differential proteins between the proteome subtypes. Table S3. Differential pS/T phosphosites between the phosphoproteome subtypes. Table S4. pS/T‐based INKA profiling of 42 PDAC tumors. Table S5. pY‐based INKA profiling of 33 PDAC tumors. Table S6. Mutation sequencing panel of frequently mutated genes in 42 PDAC tumors. Table S7. Differential analysis of pS/T sites between KRAS G12D mutated and the rest of tumors (G12V, G12R). Table S8. Differential analysis of pS/T sites between TP53 mutated and wildtype tumors. Table S9. Differential analysis of pY sites between TP53 mutated and wildtype tumors. Table S10. Differential analysis of pS/T sites between long (n = 3) and short survival (n = 3) patients. Table S11. Differential analysis of pY sites between long (n = 3) and short survival (n = 3) patients. [file MOL2-18-2020-s001.zip › mol213625-sup-0001-Fig S1.tif]

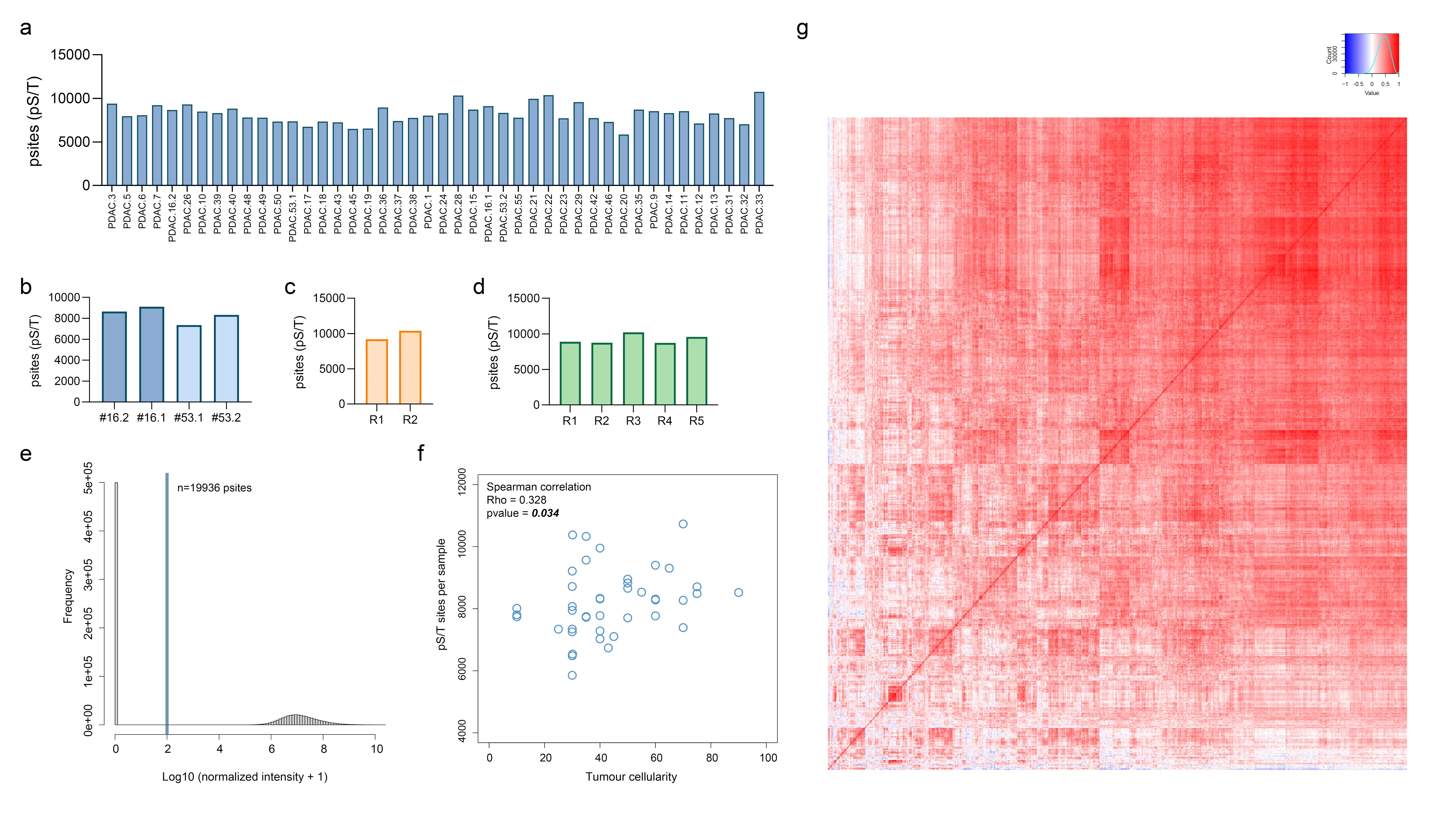

Supplement: Supplementary file 1 — Fig. S1. Large‐scale proteomic and phosphoproteomic analysis of pancreatic ductal adenocarcinoma. Fig. S2. Data overview of the pS/T phosphoproteome of pancreatic ductal adenocarcinoma. Fig. S3. Data overview of the pY phosphoproteome of pancreatic ductal adenocarcinoma. Fig. S4. Data overview of the proteome of pancreatic ductal adenocarcinoma. Fig. S5. Consensus clustering analyses of proteome and phosphoproteome. Fig. S6. Comprehensive analysis of PDAC (phospho)proteome subtypes. Fig. S7. Overall survival analysis of subtypes based on mRNA, proteome and phosphoproteome. Fig. S8. Differential kinase activities among the identified PDAC phosphoproteome subtypes. Fig. S9. Phosphoproteomic signatures among KRAS alleles G12D, G12V and G12R. Fig. S10. Association of TP53 gene mutations with pY phosphoproteome. Fig. S11. Phosphoproteome differences in short and long survival (treatment naïve) patients. Table S1. Clinicopathological characteristics of the subset (42/90) of SPACIOUS cohort tumors. Table S2. Differential proteins between the proteome subtypes. Table S3. Differential pS/T phosphosites between the phosphoproteome subtypes. Table S4. pS/T‐based INKA profiling of 42 PDAC tumors. Table S5. pY‐based INKA profiling of 33 PDAC tumors. Table S6. Mutation sequencing panel of frequently mutated genes in 42 PDAC tumors. Table S7. Differential analysis of pS/T sites between KRAS G12D mutated and the rest of tumors (G12V, G12R). Table S8. Differential analysis of pS/T sites between TP53 mutated and wildtype tumors. Table S9. Differential analysis of pY sites between TP53 mutated and wildtype tumors. Table S10. Differential analysis of pS/T sites between long (n = 3) and short survival (n = 3) patients. Table S11. Differential analysis of pY sites between long (n = 3) and short survival (n = 3) patients. [file MOL2-18-2020-s001.zip › mol213625-sup-0002-FigS2.tif]

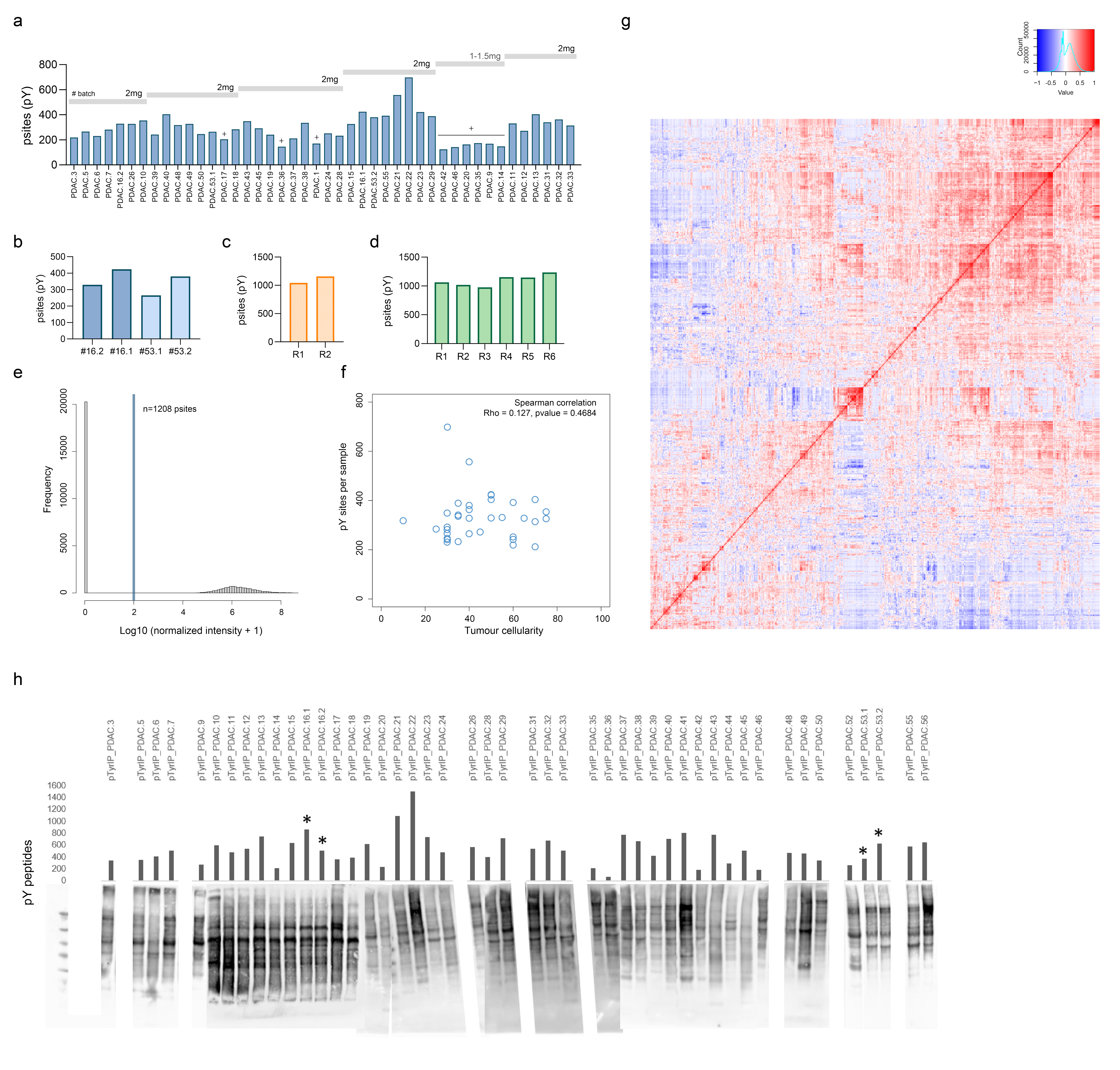

Supplement: Supplementary file 1 — Fig. S1. Large‐scale proteomic and phosphoproteomic analysis of pancreatic ductal adenocarcinoma. Fig. S2. Data overview of the pS/T phosphoproteome of pancreatic ductal adenocarcinoma. Fig. S3. Data overview of the pY phosphoproteome of pancreatic ductal adenocarcinoma. Fig. S4. Data overview of the proteome of pancreatic ductal adenocarcinoma. Fig. S5. Consensus clustering analyses of proteome and phosphoproteome. Fig. S6. Comprehensive analysis of PDAC (phospho)proteome subtypes. Fig. S7. Overall survival analysis of subtypes based on mRNA, proteome and phosphoproteome. Fig. S8. Differential kinase activities among the identified PDAC phosphoproteome subtypes. Fig. S9. Phosphoproteomic signatures among KRAS alleles G12D, G12V and G12R. Fig. S10. Association of TP53 gene mutations with pY phosphoproteome. Fig. S11. Phosphoproteome differences in short and long survival (treatment naïve) patients. Table S1. Clinicopathological characteristics of the subset (42/90) of SPACIOUS cohort tumors. Table S2. Differential proteins between the proteome subtypes. Table S3. Differential pS/T phosphosites between the phosphoproteome subtypes. Table S4. pS/T‐based INKA profiling of 42 PDAC tumors. Table S5. pY‐based INKA profiling of 33 PDAC tumors. Table S6. Mutation sequencing panel of frequently mutated genes in 42 PDAC tumors. Table S7. Differential analysis of pS/T sites between KRAS G12D mutated and the rest of tumors (G12V, G12R). Table S8. Differential analysis of pS/T sites between TP53 mutated and wildtype tumors. Table S9. Differential analysis of pY sites between TP53 mutated and wildtype tumors. Table S10. Differential analysis of pS/T sites between long (n = 3) and short survival (n = 3) patients. Table S11. Differential analysis of pY sites between long (n = 3) and short survival (n = 3) patients. [file MOL2-18-2020-s001.zip › mol213625-sup-0003-FigS3.tif]

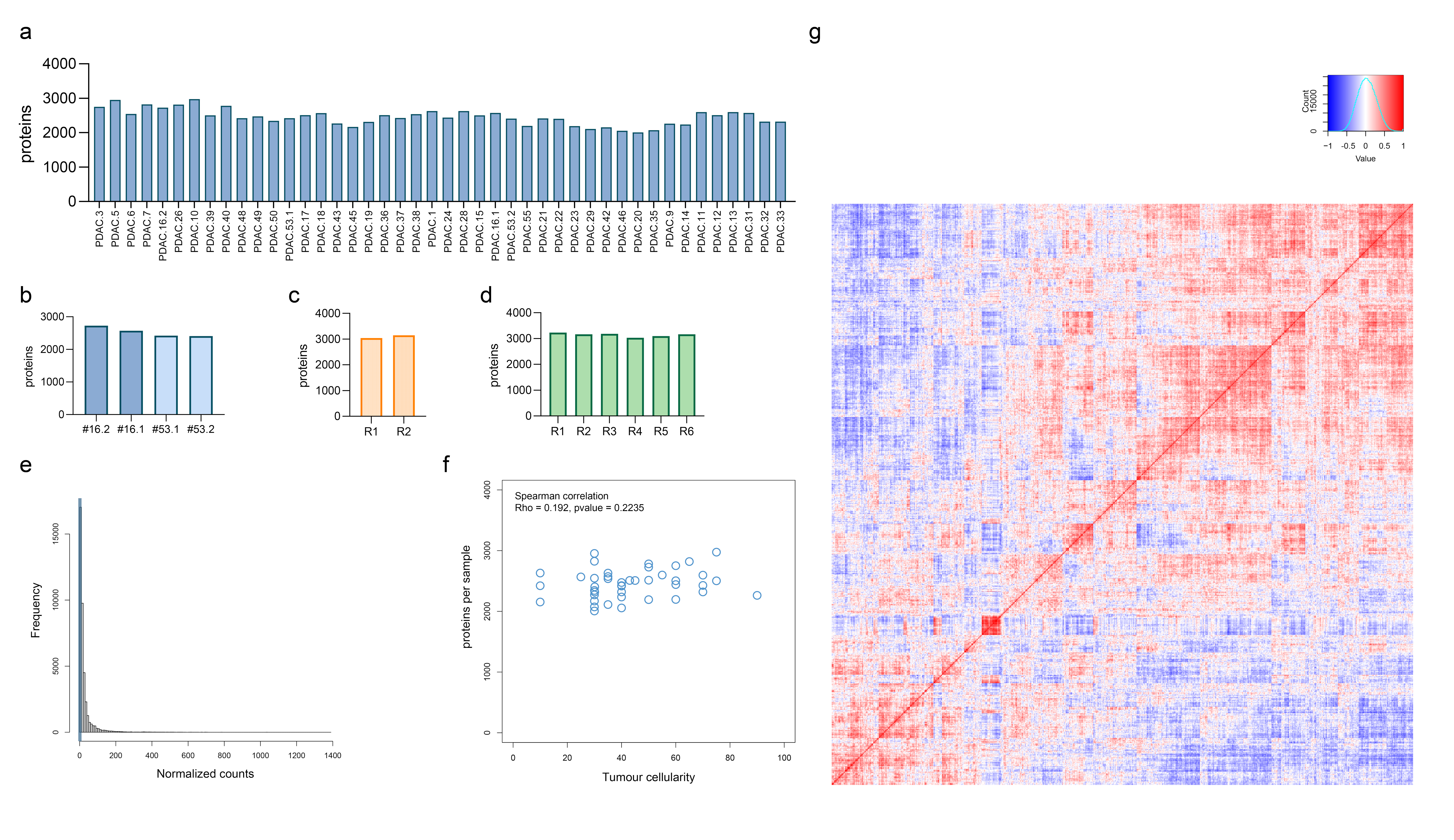

Supplement: Supplementary file 1 — Fig. S1. Large‐scale proteomic and phosphoproteomic analysis of pancreatic ductal adenocarcinoma. Fig. S2. Data overview of the pS/T phosphoproteome of pancreatic ductal adenocarcinoma. Fig. S3. Data overview of the pY phosphoproteome of pancreatic ductal adenocarcinoma. Fig. S4. Data overview of the proteome of pancreatic ductal adenocarcinoma. Fig. S5. Consensus clustering analyses of proteome and phosphoproteome. Fig. S6. Comprehensive analysis of PDAC (phospho)proteome subtypes. Fig. S7. Overall survival analysis of subtypes based on mRNA, proteome and phosphoproteome. Fig. S8. Differential kinase activities among the identified PDAC phosphoproteome subtypes. Fig. S9. Phosphoproteomic signatures among KRAS alleles G12D, G12V and G12R. Fig. S10. Association of TP53 gene mutations with pY phosphoproteome. Fig. S11. Phosphoproteome differences in short and long survival (treatment naïve) patients. Table S1. Clinicopathological characteristics of the subset (42/90) of SPACIOUS cohort tumors. Table S2. Differential proteins between the proteome subtypes. Table S3. Differential pS/T phosphosites between the phosphoproteome subtypes. Table S4. pS/T‐based INKA profiling of 42 PDAC tumors. Table S5. pY‐based INKA profiling of 33 PDAC tumors. Table S6. Mutation sequencing panel of frequently mutated genes in 42 PDAC tumors. Table S7. Differential analysis of pS/T sites between KRAS G12D mutated and the rest of tumors (G12V, G12R). Table S8. Differential analysis of pS/T sites between TP53 mutated and wildtype tumors. Table S9. Differential analysis of pY sites between TP53 mutated and wildtype tumors. Table S10. Differential analysis of pS/T sites between long (n = 3) and short survival (n = 3) patients. Table S11. Differential analysis of pY sites between long (n = 3) and short survival (n = 3) patients. [file MOL2-18-2020-s001.zip › mol213625-sup-0004-FigS4.tif]

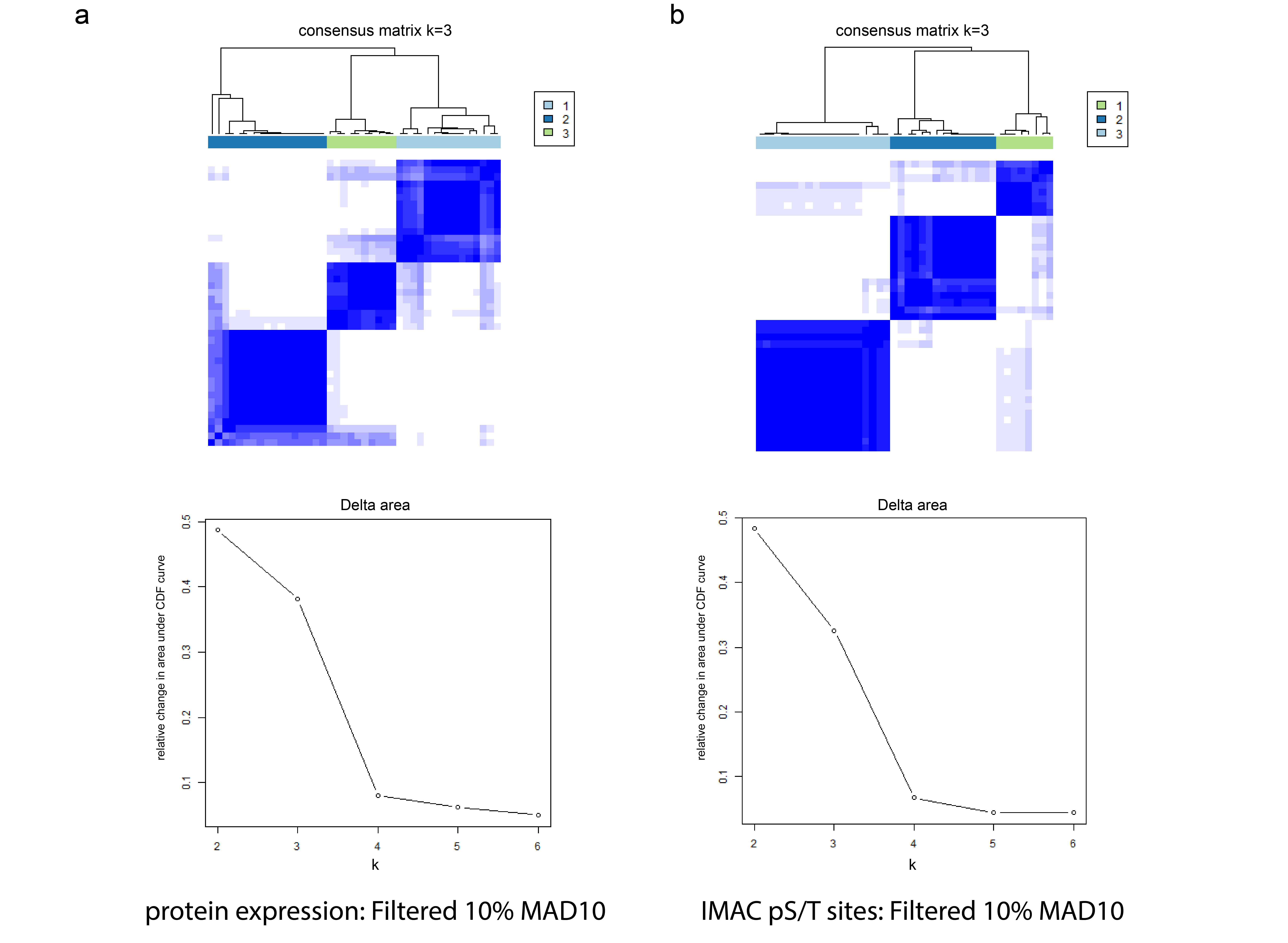

Supplement: Supplementary file 1 — Fig. S1. Large‐scale proteomic and phosphoproteomic analysis of pancreatic ductal adenocarcinoma. Fig. S2. Data overview of the pS/T phosphoproteome of pancreatic ductal adenocarcinoma. Fig. S3. Data overview of the pY phosphoproteome of pancreatic ductal adenocarcinoma. Fig. S4. Data overview of the proteome of pancreatic ductal adenocarcinoma. Fig. S5. Consensus clustering analyses of proteome and phosphoproteome. Fig. S6. Comprehensive analysis of PDAC (phospho)proteome subtypes. Fig. S7. Overall survival analysis of subtypes based on mRNA, proteome and phosphoproteome. Fig. S8. Differential kinase activities among the identified PDAC phosphoproteome subtypes. Fig. S9. Phosphoproteomic signatures among KRAS alleles G12D, G12V and G12R. Fig. S10. Association of TP53 gene mutations with pY phosphoproteome. Fig. S11. Phosphoproteome differences in short and long survival (treatment naïve) patients. Table S1. Clinicopathological characteristics of the subset (42/90) of SPACIOUS cohort tumors. Table S2. Differential proteins between the proteome subtypes. Table S3. Differential pS/T phosphosites between the phosphoproteome subtypes. Table S4. pS/T‐based INKA profiling of 42 PDAC tumors. Table S5. pY‐based INKA profiling of 33 PDAC tumors. Table S6. Mutation sequencing panel of frequently mutated genes in 42 PDAC tumors. Table S7. Differential analysis of pS/T sites between KRAS G12D mutated and the rest of tumors (G12V, G12R). Table S8. Differential analysis of pS/T sites between TP53 mutated and wildtype tumors. Table S9. Differential analysis of pY sites between TP53 mutated and wildtype tumors. Table S10. Differential analysis of pS/T sites between long (n = 3) and short survival (n = 3) patients. Table S11. Differential analysis of pY sites between long (n = 3) and short survival (n = 3) patients. [file MOL2-18-2020-s001.zip › mol213625-sup-0005-FigS5.tif]

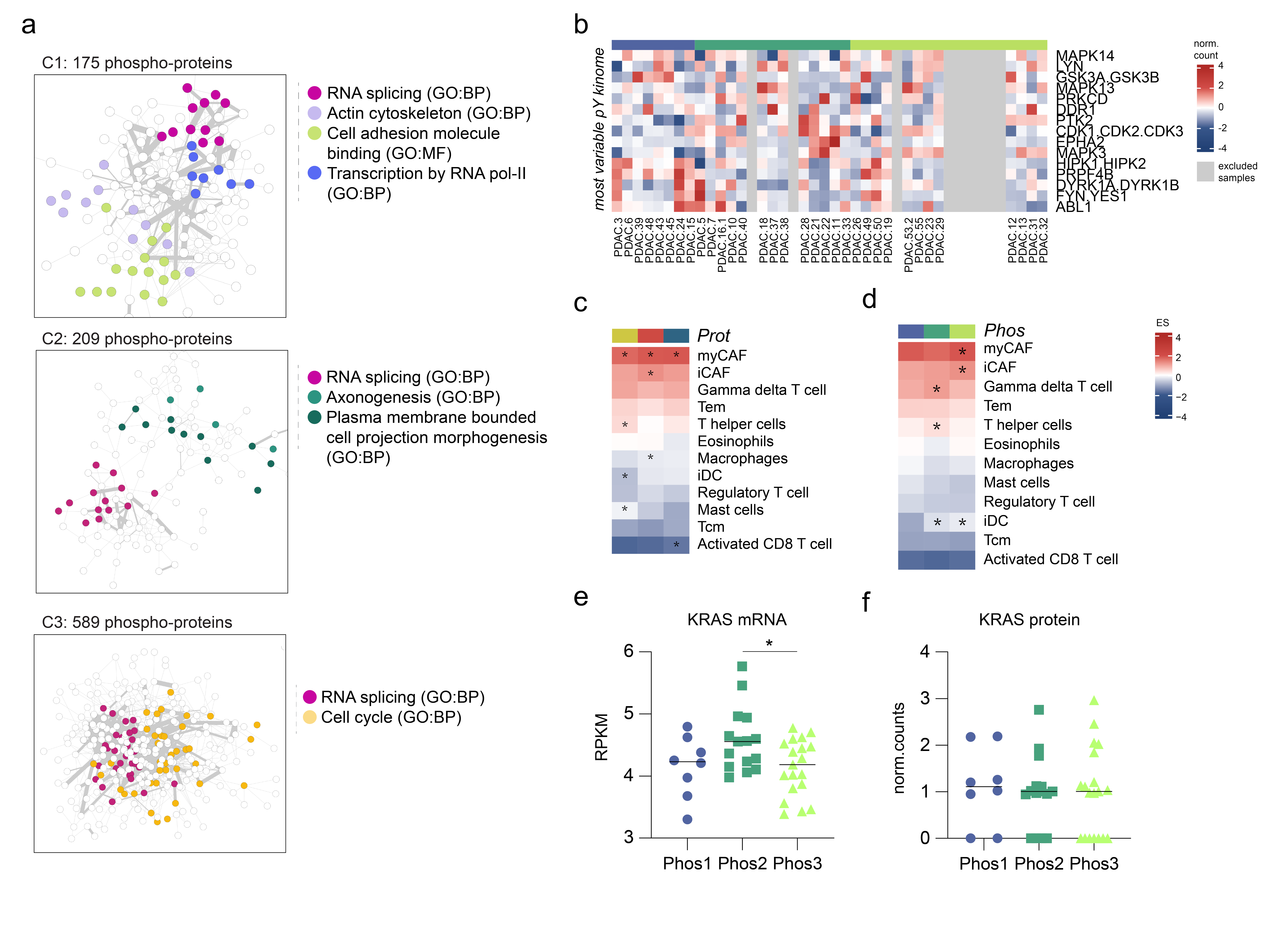

Supplement: Supplementary file 1 — Fig. S1. Large‐scale proteomic and phosphoproteomic analysis of pancreatic ductal adenocarcinoma. Fig. S2. Data overview of the pS/T phosphoproteome of pancreatic ductal adenocarcinoma. Fig. S3. Data overview of the pY phosphoproteome of pancreatic ductal adenocarcinoma. Fig. S4. Data overview of the proteome of pancreatic ductal adenocarcinoma. Fig. S5. Consensus clustering analyses of proteome and phosphoproteome. Fig. S6. Comprehensive analysis of PDAC (phospho)proteome subtypes. Fig. S7. Overall survival analysis of subtypes based on mRNA, proteome and phosphoproteome. Fig. S8. Differential kinase activities among the identified PDAC phosphoproteome subtypes. Fig. S9. Phosphoproteomic signatures among KRAS alleles G12D, G12V and G12R. Fig. S10. Association of TP53 gene mutations with pY phosphoproteome. Fig. S11. Phosphoproteome differences in short and long survival (treatment naïve) patients. Table S1. Clinicopathological characteristics of the subset (42/90) of SPACIOUS cohort tumors. Table S2. Differential proteins between the proteome subtypes. Table S3. Differential pS/T phosphosites between the phosphoproteome subtypes. Table S4. pS/T‐based INKA profiling of 42 PDAC tumors. Table S5. pY‐based INKA profiling of 33 PDAC tumors. Table S6. Mutation sequencing panel of frequently mutated genes in 42 PDAC tumors. Table S7. Differential analysis of pS/T sites between KRAS G12D mutated and the rest of tumors (G12V, G12R). Table S8. Differential analysis of pS/T sites between TP53 mutated and wildtype tumors. Table S9. Differential analysis of pY sites between TP53 mutated and wildtype tumors. Table S10. Differential analysis of pS/T sites between long (n = 3) and short survival (n = 3) patients. Table S11. Differential analysis of pY sites between long (n = 3) and short survival (n = 3) patients. [file MOL2-18-2020-s001.zip › mol213625-sup-0006-FigS6.tif]

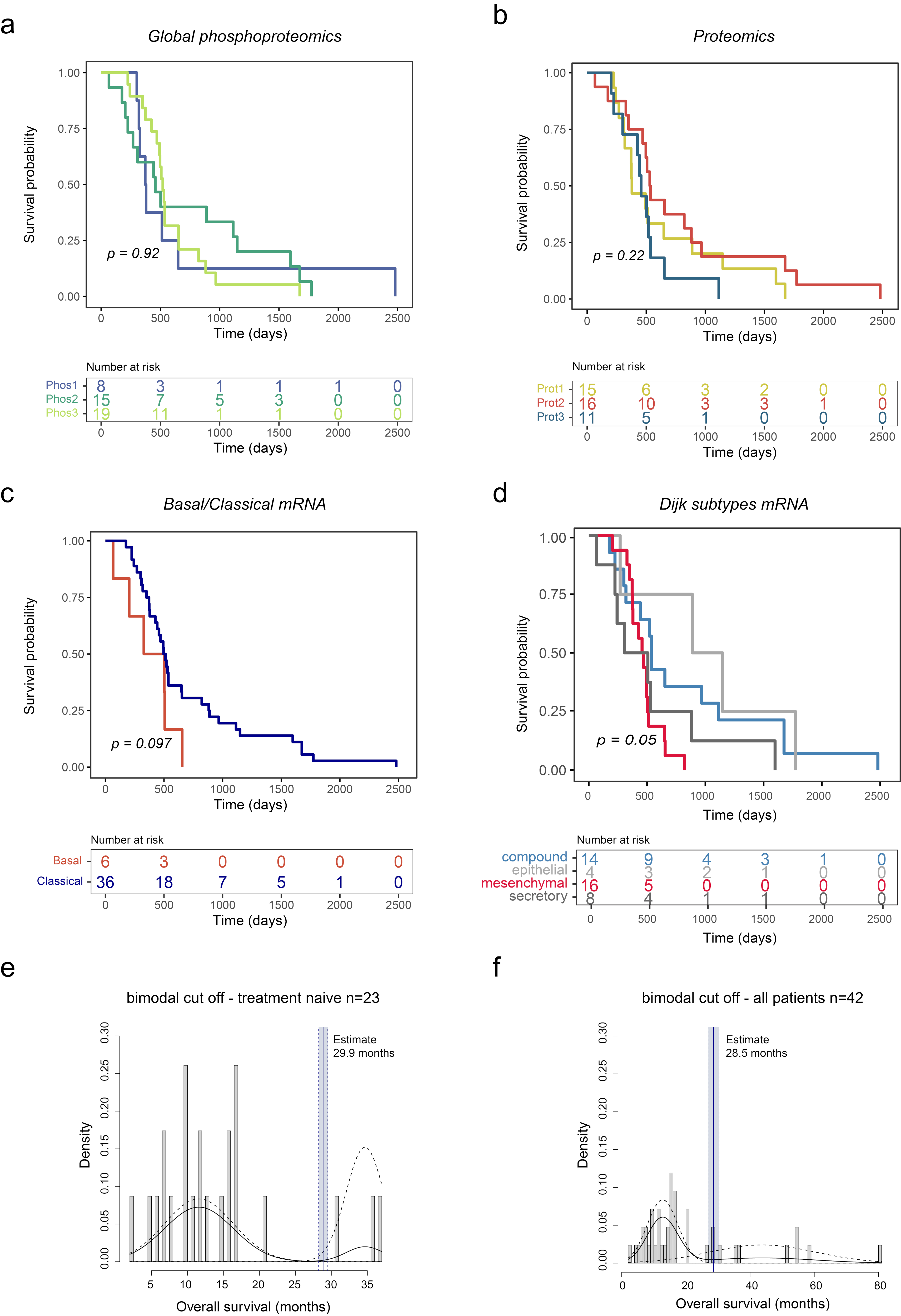

Supplement: Supplementary file 1 — Fig. S1. Large‐scale proteomic and phosphoproteomic analysis of pancreatic ductal adenocarcinoma. Fig. S2. Data overview of the pS/T phosphoproteome of pancreatic ductal adenocarcinoma. Fig. S3. Data overview of the pY phosphoproteome of pancreatic ductal adenocarcinoma. Fig. S4. Data overview of the proteome of pancreatic ductal adenocarcinoma. Fig. S5. Consensus clustering analyses of proteome and phosphoproteome. Fig. S6. Comprehensive analysis of PDAC (phospho)proteome subtypes. Fig. S7. Overall survival analysis of subtypes based on mRNA, proteome and phosphoproteome. Fig. S8. Differential kinase activities among the identified PDAC phosphoproteome subtypes. Fig. S9. Phosphoproteomic signatures among KRAS alleles G12D, G12V and G12R. Fig. S10. Association of TP53 gene mutations with pY phosphoproteome. Fig. S11. Phosphoproteome differences in short and long survival (treatment naïve) patients. Table S1. Clinicopathological characteristics of the subset (42/90) of SPACIOUS cohort tumors. Table S2. Differential proteins between the proteome subtypes. Table S3. Differential pS/T phosphosites between the phosphoproteome subtypes. Table S4. pS/T‐based INKA profiling of 42 PDAC tumors. Table S5. pY‐based INKA profiling of 33 PDAC tumors. Table S6. Mutation sequencing panel of frequently mutated genes in 42 PDAC tumors. Table S7. Differential analysis of pS/T sites between KRAS G12D mutated and the rest of tumors (G12V, G12R). Table S8. Differential analysis of pS/T sites between TP53 mutated and wildtype tumors. Table S9. Differential analysis of pY sites between TP53 mutated and wildtype tumors. Table S10. Differential analysis of pS/T sites between long (n = 3) and short survival (n = 3) patients. Table S11. Differential analysis of pY sites between long (n = 3) and short survival (n = 3) patients. [file MOL2-18-2020-s001.zip › mol213625-sup-0007-FigS7.tif]

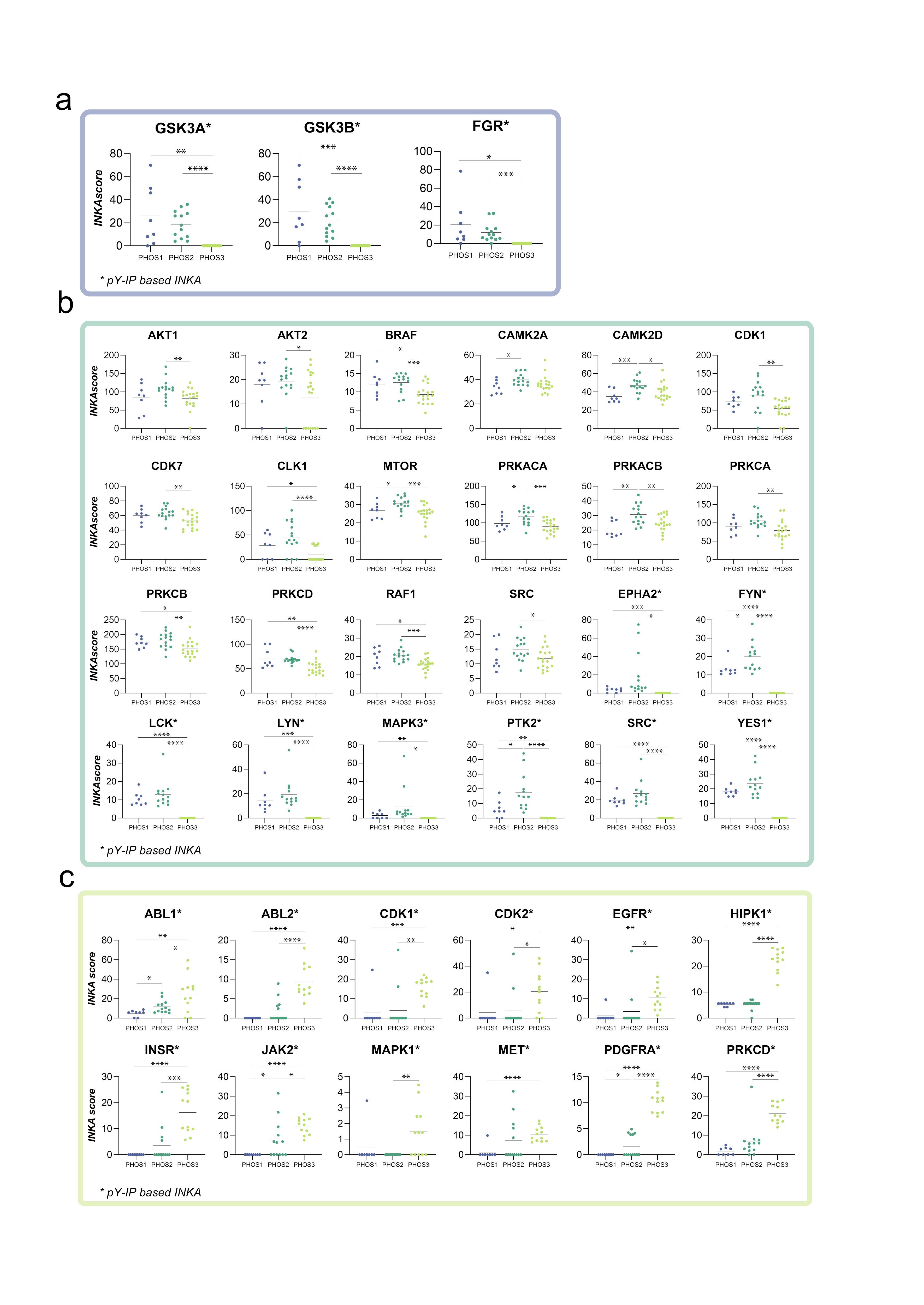

Supplement: Supplementary file 1 — Fig. S1. Large‐scale proteomic and phosphoproteomic analysis of pancreatic ductal adenocarcinoma. Fig. S2. Data overview of the pS/T phosphoproteome of pancreatic ductal adenocarcinoma. Fig. S3. Data overview of the pY phosphoproteome of pancreatic ductal adenocarcinoma. Fig. S4. Data overview of the proteome of pancreatic ductal adenocarcinoma. Fig. S5. Consensus clustering analyses of proteome and phosphoproteome. Fig. S6. Comprehensive analysis of PDAC (phospho)proteome subtypes. Fig. S7. Overall survival analysis of subtypes based on mRNA, proteome and phosphoproteome. Fig. S8. Differential kinase activities among the identified PDAC phosphoproteome subtypes. Fig. S9. Phosphoproteomic signatures among KRAS alleles G12D, G12V and G12R. Fig. S10. Association of TP53 gene mutations with pY phosphoproteome. Fig. S11. Phosphoproteome differences in short and long survival (treatment naïve) patients. Table S1. Clinicopathological characteristics of the subset (42/90) of SPACIOUS cohort tumors. Table S2. Differential proteins between the proteome subtypes. Table S3. Differential pS/T phosphosites between the phosphoproteome subtypes. Table S4. pS/T‐based INKA profiling of 42 PDAC tumors. Table S5. pY‐based INKA profiling of 33 PDAC tumors. Table S6. Mutation sequencing panel of frequently mutated genes in 42 PDAC tumors. Table S7. Differential analysis of pS/T sites between KRAS G12D mutated and the rest of tumors (G12V, G12R). Table S8. Differential analysis of pS/T sites between TP53 mutated and wildtype tumors. Table S9. Differential analysis of pY sites between TP53 mutated and wildtype tumors. Table S10. Differential analysis of pS/T sites between long (n = 3) and short survival (n = 3) patients. Table S11. Differential analysis of pY sites between long (n = 3) and short survival (n = 3) patients. [file MOL2-18-2020-s001.zip › mol213625-sup-0008-FigS8.tif]

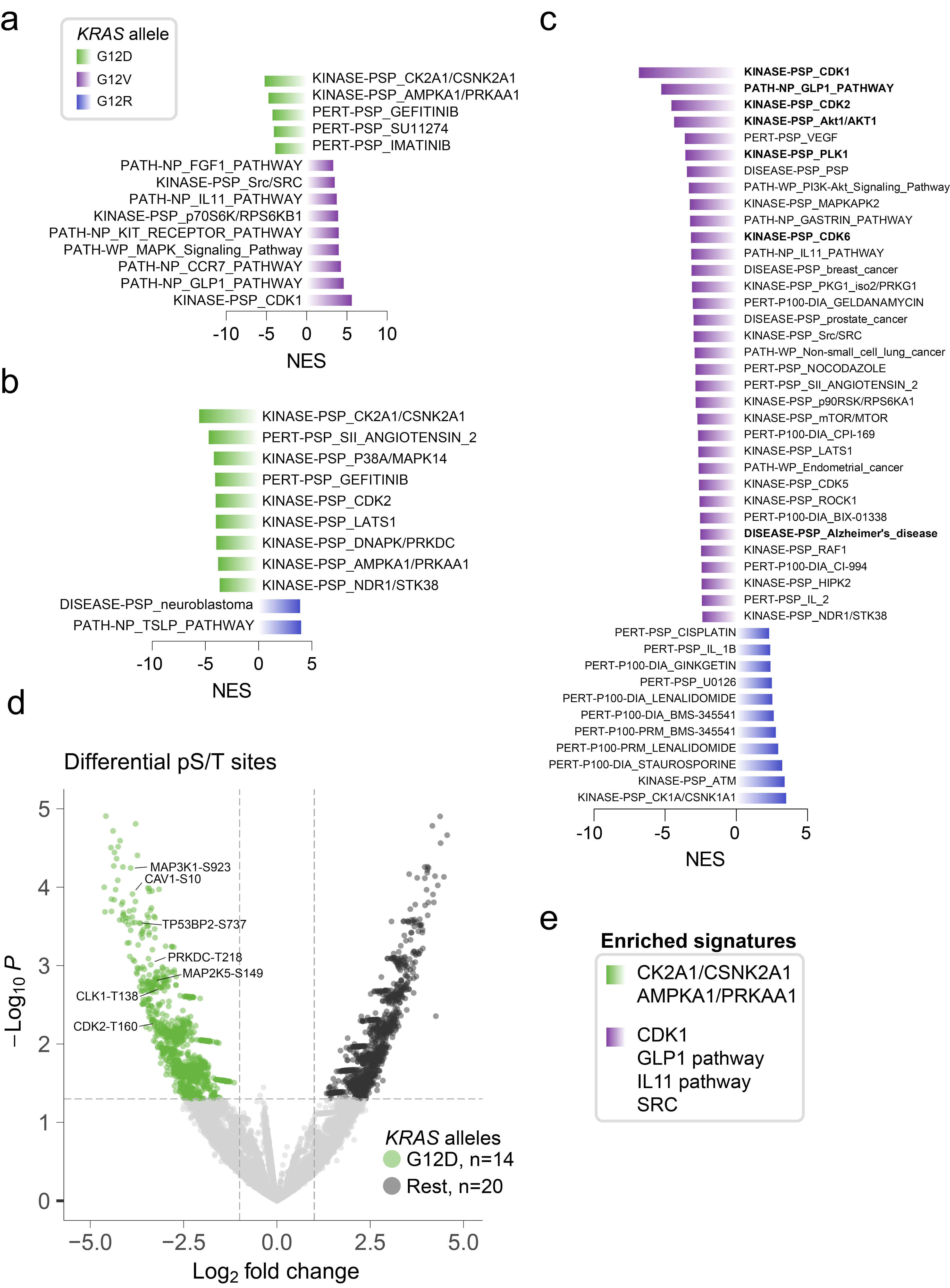

Supplement: Supplementary file 1 — Fig. S1. Large‐scale proteomic and phosphoproteomic analysis of pancreatic ductal adenocarcinoma. Fig. S2. Data overview of the pS/T phosphoproteome of pancreatic ductal adenocarcinoma. Fig. S3. Data overview of the pY phosphoproteome of pancreatic ductal adenocarcinoma. Fig. S4. Data overview of the proteome of pancreatic ductal adenocarcinoma. Fig. S5. Consensus clustering analyses of proteome and phosphoproteome. Fig. S6. Comprehensive analysis of PDAC (phospho)proteome subtypes. Fig. S7. Overall survival analysis of subtypes based on mRNA, proteome and phosphoproteome. Fig. S8. Differential kinase activities among the identified PDAC phosphoproteome subtypes. Fig. S9. Phosphoproteomic signatures among KRAS alleles G12D, G12V and G12R. Fig. S10. Association of TP53 gene mutations with pY phosphoproteome. Fig. S11. Phosphoproteome differences in short and long survival (treatment naïve) patients. Table S1. Clinicopathological characteristics of the subset (42/90) of SPACIOUS cohort tumors. Table S2. Differential proteins between the proteome subtypes. Table S3. Differential pS/T phosphosites between the phosphoproteome subtypes. Table S4. pS/T‐based INKA profiling of 42 PDAC tumors. Table S5. pY‐based INKA profiling of 33 PDAC tumors. Table S6. Mutation sequencing panel of frequently mutated genes in 42 PDAC tumors. Table S7. Differential analysis of pS/T sites between KRAS G12D mutated and the rest of tumors (G12V, G12R). Table S8. Differential analysis of pS/T sites between TP53 mutated and wildtype tumors. Table S9. Differential analysis of pY sites between TP53 mutated and wildtype tumors. Table S10. Differential analysis of pS/T sites between long (n = 3) and short survival (n = 3) patients. Table S11. Differential analysis of pY sites between long (n = 3) and short survival (n = 3) patients. [file MOL2-18-2020-s001.zip › mol213625-sup-0009-FigS9.tif]
